# Scalable production and immunogenicity of a cholera conjugate vaccine

**DOI:** 10.1016/j.vaccine.2021.10.005

**Published:** 2021-11-16

**Authors:** Suhi Jeon, Meagan Kelly, Jeesun Yun, Byungman Lee, Minchul Park, Yoonhee Whang, Chankyu Lee, Yuan-Di Halvorsen, Smriti Verma, Richelle C. Charles, Jason B. Harris, Stephen B. Calderwood, Daniel T. Leung, Taufiqur R. Bhuiyan, Firdausi Qadri, Mohammad Kamruzzaman, Somyoung Cho, Willie F. Vann, Peng Xu, Pavol Kováč, Ravi Ganapathy, Julia Lynch, Edward T. Ryan

**Affiliations:** a Eubiologics Ltd, Gangnam-gu, Seoul, South Korea; b Division of Infectious Diseases, Massachusetts General Hospital, Boston, MA, USA; c Department of Medicine, Harvard Medical School, Boston, MA, USA; d Department of Pediatrics, Harvard Medical School, Boston, MA, USA; e Division of Global Health, MassGeneral Hospital for Children, Boston, MA, USA; f Division of Infectious Diseases, University of Utah School of Medicine, Salt Lake City, UT, USA; g icddr,b (International Centre for Diarrhoeal Disease Research, Bangladesh), Dhaka, Bangladesh; h International Vaccine Institute, Seoul, South Korea; i Center for Biologics Evaluation and Research, U.S. Food and Drug Administration, Silver Spring, MD, USA; j NIDDK, LBC, National Institutes of Health, Bethesda, MD, USA; k Department of Immunology and Infectious Diseases, Harvard T.H. Chan School of Public Health, Boston, MA, USA

**Keywords:** Cholera conjugate vaccine, *Vibrio cholerae*, O-specific polysaccharide, OSP: rTTHc

## Abstract

There is a need to develop cholera vaccines that are protective in young children under 5 years of age, which induce long-term immunity, and which can be incorporated into the Expanded Programme of Immunization (EPI) in cholera-endemic countries. The degree of protection afforded by currently available oral cholera vaccines (OCV) to young children is significantly lower than that induced by vaccination of older vaccine recipients. Immune responses that protect against cholera target the O-specific polysaccharide (OSP) of *Vibrio cholerae*, and young children have poor immunological responses to bacterial polysaccharides, which are T cell independent antigens. To overcome this, we have developed a cholera conjugate vaccine (CCV) containing the OSP of *V. cholerae* O1, the main cause of endemic and epidemic cholera. Here, we describe production of CCV through a scalable manufacturing process and preclinical evaluation of immunogenicity in the presence and absence of aluminum phosphate (alum) as an adjuvant. The vaccine displays *V. cholerae* O1 Inaba OSP in sun-burst display via single point attachment of core oligosaccharide to a recombinant tetanus toxoid heavy chain fragment (rTTHc). Two different pilot-scale production batches of non-GMP CCV were manufactured and characterized in terms of physico-chemical properties and immunogenicity. In preclinical testing, the vaccine induced OSP- and lipopolysaccharide (LPS)-specific IgG and IgM responses, vibriocidal responses, memory B cell responses, and protection in a *V. cholerae* O1 challenge model. The addition of alum to the administered vaccine increased OSP-specific immune responses. These results support evaluation of CCV in humans.

## 1 Introduction

Approximately 3–5 million humans develop cholera each year, with millions more at risk, and cholera kills tens of thousands of humans annually [1], [2]. Cholera is endemic in over 50 countries, and we are currently in the 6th decade of the most recent cholera pandemic, with little evidence of abatement. The Global Task Force on Cholera Control (GTFCC) has endorsed a “Road Map for Ending Cholera by 2030” that includes use of cholera vaccine with the objective to reduce cholera deaths by 90% worldwide, and eliminate cholera in at least 20 countries by 2030 (https://www.who.int/cholera/publications/global-roadmap.pdf) [3], [4]. Unfortunately, a number of factors make attaining the 2030 goal challenging. First, only tens of millions of doses of cholera vaccine are currently being produced globally each year, despite the fact that WHO estimates that 1.5 billion people are at risk of cholera [3]. Second, the currently available WHO-prequalified oral cholera vaccines (OCV) require two-doses administered 14 days apart and are typically delivered through resource-intensive mass vaccination campaigns. Under current recommendations, these campaigns must be repeated every three to five years in at-risk communities because of the limited duration of protection afforded by OCV. OCVs are not recommended for delivery through the routine Expanded Programme of Immunization (EPI) as they are poorly protective against cholera in young children under 5 years of age [5], [6], [7], [8], [9], despite children bearing a large global burden of cholera [3], [10], [11]. Many stakeholders, including the GTFCC and Gavi-the Vaccine Alliance, have called for development of new or improved cholera vaccines, especially those that provide durable protection of young children under 5 years of age, and that can be incorporated into EPI schedules [12], [13], [14].

Protection against cholera is largely mediated by antibody responses that target the O-specific polysaccharide (OSP) component of *Vibrio cholerae* lipopolysaccharide (LPS) [15], [16]. OSP is a T cell-independent antigen, and immune responses in young children to T cell-independent antigens are often poor. Conjugating polysaccharides to carrier proteins (as in a conjugate vaccine) induces more prominent and long-lasting polysaccharide-specific immune responses in young children [17], [18], [19], [20], [21], [22]. Parenteral cholera vaccines (containing killed *V. cholerae* organisms/unconjugated OSP) were used for decades to protect against cholera, providing 40–70% protective efficacy, including in children under 5 years of age [23], [24], [25], [26], [27], [28], [29], but their use fell off due to the need for frequent re-boosting. To more directly target induction of durable OSP-specific responses, we developed a prototype cholera conjugate vaccine (CCV) [30]. This vaccine includes purified OSP from *V. cholerae* O1 El Tor Inaba strain PIC018 conjugated to a recombinant tetanus toxoid heavy chain fragment (rTTHc) [30]. The resultant product was immunogenic and protective in preclinical animal models [30]. Here, we report revision of production protocols of CCV to a scalable manufacturing process, evaluation of immunoreactivity of the conjugate using convalescent phase samples from humans surviving cholera, and preclinical immunogenicity evaluation of CCV.

## 2 Materials and methods

### 2.1 Bacterial strains and reagents

Production of CCV was advanced from previous bench-top non-scalable production [30]; previously produced OSP from *V. cholerae* O1 El Tor Inaba strain PIC018 and recombinantly expressed rTTHc from *Escherichia coli* were used as initial production standards. For immunologic assays, OSP was conjugated to bovine serum albumin (BSA Sigma) as previously described [30]. LPS for use in immunologic assays was also prepared from *V. cholerae* O1 PIC018 as previously described [31].

### 2.2 Production of OSP-rTTHc

Production of Inaba O-specific polysaccharide (OSP), as an integral component of lipopolysaccharide (LPS) in *Vibrio cholerae* strains, involved developing a *V. cholerae* O1 El Tor PIC018 seed-inoculum in shake-flasks in semi-defined medium containing salts, yeast extract (10.0 g/L, BectonDickinson), amino acids (L-tryptophan, 0.05 g/L, Merck; Casamino acid, 30.0 g/L; Solabia) and antibiotic (streptomycin, 100 µg/L, TEKnova), followed by inoculation of seed culture into a production fermenter with the same medium. A feed strategy was used to increase cell density using medium containing sugar, salts, yeast extract, amino acids and antibiotic. Following *V. cholerae* O1 Inaba fermentation, acid-hydrolysis was performed after cell concentration and washing by microfiltration/diafiltration (0.2 µm Hydrosart MF/DF 0.6 m^2^ membrane, Sartorius) in tangential flow filtration (TFF) mode; and cellular debris was removed by centrifugation (17,000 rpm) and 0.2 µm filtration. OSP purification then involved application of 30 kDa cut-off membrane cassettes (Sartocon Cassette PES 0.7 m^2^, 30 kDa, Sartorius) in TFF mode; and permeate was recovered and processed again through 5 kDa cut-off membrane cassettes (Sartocon Cassette Hydrosart 0.7 m^2^, 5 kDa, Sartorius) in TFF mode, with the retentate from the latter step concentrated and diafiltered into final buffer followed by sterile-filtration using a 0.2 µm filter. Final purified OSP was analyzed for purity via SE-HPLC (size exclusion -high performance liquid chromatography; G2000SWxL TSKgel), polysaccharide content via resorcinol assay, and protein content via BCA (bicinchoninic acid) assay.

Production of rTTHc involved developing a recombinant *Escherichia coli-*rTTHc strain seed-inoculum in shaker-flasks containing APS-LB (Alternate Protein Source-Luria-Bertani) medium (a semi-defined medium containing LB medium components plus ammonium chloride [6.0 g/L, Merck], phosphate [sodium dihydrogen phosphate monohydrate 0.5 g/L, potassium phosphate dibasic 1.0 g/L, di-potassium hydrogen phosphate 3.0 g/L, Merck] and peptone [30.0 g/L, VWR]) and an antibiotic, and inoculation of seed culture into a production fermenter with the same medium. A feed strategy was used to increase cell density using medium containing sugar, yeast extract and peptone. Target protein induction was done using isopropyl β-D-1-thiogalactopyranoside (IPTG; 0.012 g/L, Enzo) and galactose (D-Galactose,15.0 g/L, BD DIFCO) combination strategy using an auto-induction mechanism. The fermenter culture was harvested by centrifugation (17,000 rpm) once the depletion of feed nutrient was observed. The harvested cell paste was frozen at –70 ± 10 °C until used for rTTHc isolation and purification. rTTHc was purified from supernatant obtained after lysis of cell paste. rTTHc purification involved Ion-Exchange (Q Sepharose XL, Cytiva) and Hydrophobic Interaction (Butyl Sepharose HP, Cytiva) chromatography steps, as well as a TFF step (10 kDa Hydrosart, Sartorius). Final purified rTTHc was analyzed for purity via SEC-HPLC (GEL G2000 SWXL, TSK), sodium dodecyl sulfate–polyacrylamide gel electrophoresis (SDS-PAGE) and Western blot analysis, and protein content via BCA assay.

**Fig. 1:**
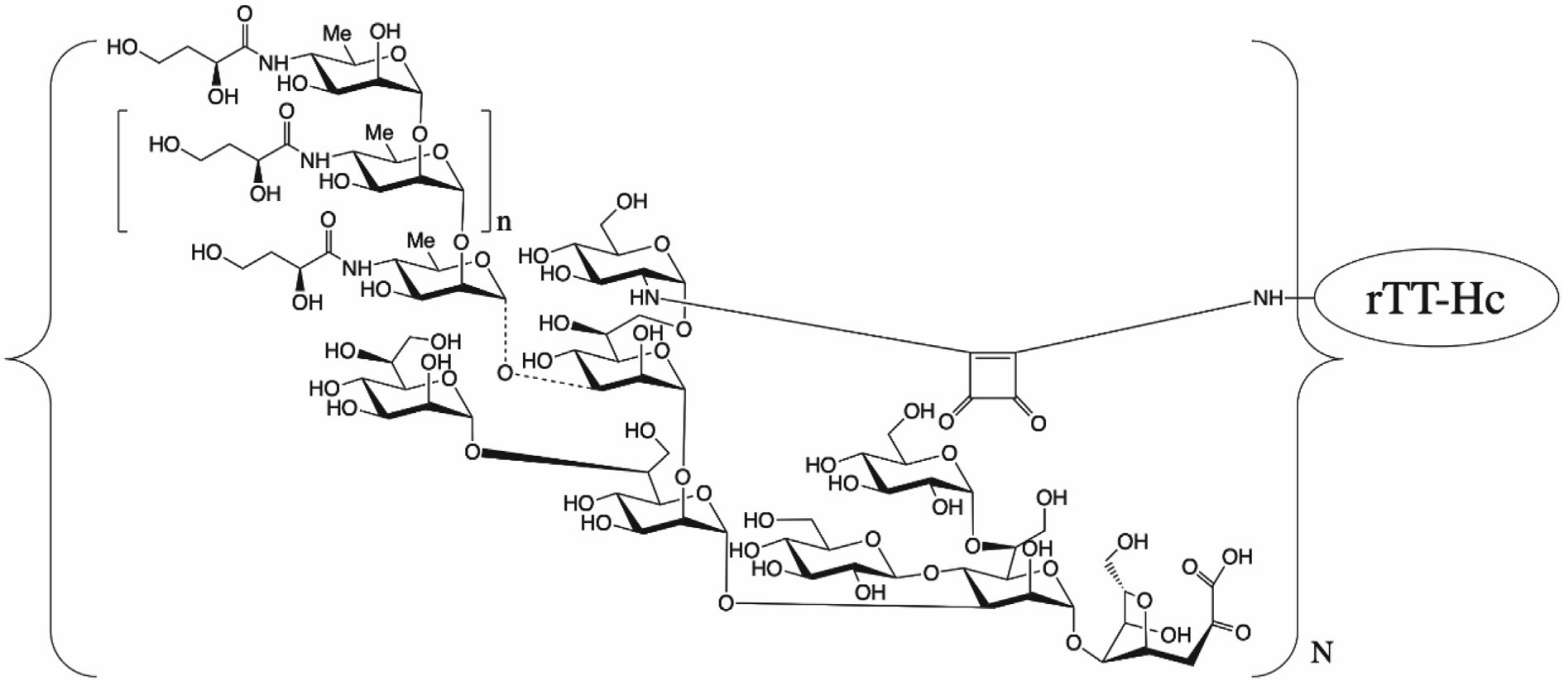
**Schematic of OSP:rTTHc cholera conjugate vaccine**. *V. cholerae* O1 OSP is comprised of repetitive (n = ∼7–22) perosamines attached to an oligosaccharide core that contains a glucosamine that can be used to link polysaccharide to a protein carrier using squarate chemistry. Such linkage is via single point attachment resulting in a “sun burst display” of OSP. Various molar loadings (N) of polysaccharide to protein carrier can be produced. rTTHc: recombinant tetanus toxin heavy chain fragment (52 kDa); OSP: O-specific polysaccharide (∼6 kDa). Adapted from Sayeed et al [30].

For making CCV conjugate (Fig. 1), purified OSP was concentrated and diafiltered into 50 mM phosphate buffer using 5 kDa TFF cassettes to a final concentration of ≥ 50 mg/mL. After dimethyl squarate ester (DSE, 3,4-dimethoxy-3-cyclobutene-1,2-dione) derivatization (labeling reaction) of OSP, extra DSE and hydrolyzed DSE were removed by diafiltration. Purified rTTHc protein was concentrated to ≥ 30 mg/mL and diafiltered into 50 mM sodium borate buffer (pH 9.8) using 10 kDa TFF cassettes. The concentrated rTTHc was combined with ∼ 15 M equivalents of aqueous OSP-squarate by slow mixing at ∼ 100 rpm and incubated at 22 ± 2 °C for 24 hrs, with mixing at ∼ 200 rpm. After completion of the reaction, the final OSP-rTTHc conjugate was diafiltered into 10 mM ammonium carbonate buffer (pH 9.1) using 30 kDa TFF cassettes targeting a final concentration of OSP in the conjugate of ≥ 400 µg/mL. After undergoing 0.2 μm filtration, the CCV product was stored at 2–8 °C until use. Final OSP:rTTHc was analyzed via SEC-HPLC, polysaccharide content via resorcinol assay, protein content via BCA assay, and SELDI-TOF-MS (surface-enhanced laser desorption/ionization-time of flight-mass spectrometry) analysis to determine average molecular weight (M.w.) of OSP and molar ratio of OSP to rTTHc in final conjugates.

To assess reproducibility and production parameters, two batches of CCV were made: EubB2006-1 and EubB2006-2. To produce EubB2006-1, the reaction involved mixing 1.7 g of purified OSP, labelled with DSE and concentrated to ∼ 50 mg/mL, with 0.8 g of the concentrated rTTHc (30 mg/mL) in 50 mM sodium borate buffer (pH 9.8) and made into a final volume of 65 mL. This entailed a starting OSP concentration of 26 mg/mL. To produce EubB2006-2, the reaction involved mixing 1.7 g of the purified OSP, labelled with DSE and concentrated to ∼ 50 mg/mL, with 0.8 g of the concentrated rTTHc (30 mg/mL) in 50 mM sodium borate buffer (pH 9.8) and made into a final volume of 130 mL. This entailed a starting OSP concentration of 13 mg/mL.

### 2.3 Immunoreactivity of conjugates using human serum

To assess immune-recognition of the OSP display on CCV, we performed antigen-specific ELISAs using pooled sera collected from humans with cholera in Bangladesh, and compared these responses to controls of humans with typhoid fever (n = 5) in Bangladesh. We separately assessed immune responses in humans with cholera caused by *V. cholerae* O1 Inaba serotype (n = 5) versus Ogawa serotype (n = 5). We coated wells (100 ng/well) with either OSP:rTTHc EubB2006-1, OSP:rTTHc EubB2006-2, OSP:BSA, or rTTHc, and assessed immunoreactivity via ELISA using human sera (1:250 dilution) as previously described [30]. We compared responses in acute phase samples (day 2) to convalescent phase samples (day 7). Following incubation, plates were washed and developed with a 0.55 mg/mL solution of 2,2ʹ 0-azinobis (3-ethylbenzothiazoline-6-sulfonic acid) (ABTS; Sigma) with 0.03% H_2_O_2_ (Sigma), and optical density at 405 nm was determined with a Vmax microplate kinetic reader (Molecular Devices Corp.). Plates were read for 5 min. at 30 s intervals, and the maximum slope for an optical density change of 0.2 U was reported as millioptical density units per minute (mOD/min.) [30], [31].

## 3 Immunogenicity

### 3.1 Vaccination and collection of samples

We immunized female Swiss-Webster mice (3–5 weeks old; Charles River Laboratories) with 10 µg of polysaccharide per dose of OSP:rTTHc of EubB2006-1 or EubB2006-2. A separate cohort of mice was immunized with EubB2006-1 with aluminum phosphate (Adju-Phos, InvivoGen, San Diego, CA), mixed in a 1:1 volume per manufacturers’ instruction. Control cohorts included mice vaccinated with aluminum phosphate alone, or Phosphate Buffered Saline (PBS) alone. To assess kinetics of immune responses, mice were vaccinated intramuscularly on days 0, 14, 28, and then again on day 56. We collected blood samples via tail bleeding on days 0, 7, 14, 21, 28, 35, 56, 63 and at sacrifice. Samples were collected, processed, aliquoted, and stored as previously described [31], [32], [33]. To assess memory B cell responses, we isolated splenocytes at the time of sacrifice and processed cells for ELISPOT analysis as previously described [31].

### 3.2 Antigen-specific antibody responses in serum

We assessed OSP and TT-specific IgG and IgM responses in serum using standard enzyme-linked immunosorbent assay (ELISA) protocols as previously described (rTTHc-specific responses at IgG 1:1000 dilution; IgM at 1:25 dilution; OSP-specific responses at 1:25 IgG and IgM) [30], [31], [32]. Briefly, plates were incubated at 37 °C for 1.5 h and then washed via multichannel pipette with PBS-T (0.05%). 0.1 mL detection antibody solution was then applied: horseradish peroxidase-conjugated to anti-mouse IgG or IgM antibody (diluted 1:1000 in 0.1% BSA in PBS-Tween) (Southern Biotech). Plates were read in a Vmax microplate kinetic reader (Molecular Devices Corp.) as described above. Plates were normalized using control sera. We defined a responder as having a post-vaccination ELISA unit greater than two times the highest value in day 63 samples from control mice vaccinated with PBS.

### 3.3 Serum vibriocidal responses

We assessed serum vibriocidal antibody titers against *V. cholerae* O1 El Tor Inaba strain PIC018 as previously described [30], [31]. Vibriocidal titer was calculated as the dilution of serum causing 50% reduction in optical density compared to control wells without serum. We defined responders as having at least a 4-fold increase in vibriocidal titer compared to pre-vaccination.

### 3.4 Memory B cell responses

Nitrocellulose bottom plates (MAHAS4510, Millipore) were coated with OSP:BSA (100 ng/well), or keyhole limpet hemocyanin (KLH; Pierce Biotechnology) (2.5 μg/ml, negative control) or goat anti-mouse IgG (Southern Biotech) [30], [31], [34]. We assessed memory B cell responses after the final round of immunization as previously described [30], [31], [34]. We used unstimulated samples as negative controls and subtracted responses to KLH as a control for the plate. We assessed total IgG secreting cells, as well as OSP-specific IgG cells by ELISPOT assay [30], [31]. We defined responders as having detectable OSP-specific IgG cells per 10^5^ splenocytes at the time of sacrifice.

### 3.5 Mouse challenge model

To assess protection afforded by vaccination, we used the standard cholera neonatal mouse cholera challenge assay, as previously described [30], using wild-type *V. cholerae* O1 El Tor Inaba strain N16961 as the challenge strain. In brief, we removed 3–5 day-old un-immunized CD-1 suckling mice from dams two hours prior to inoculation. We then administered to pups a 50 μL inoculum comprised of 1x10^9^cfu of *V. cholerae* O1 El Tor Inaba strain N16961 mixed with a 1:1 dilution of pooled day 63 sera from mice intramuscularly immunized with placebo, OSP:rTTHc, or OSP:rTTHc with aluminum phosphate. Following oral challenge, we kept neonates at 30 °C and monitored animals every 3 h for 36 h, after which surviving animals were euthanized.

### 3.6 Ethics statement

Plasma samples from humans recovering from cholera caused by *V. cholerae* O1 were collected from patients at the International Centre for Diarrhoeal Disease Research in Dhaka, Bangladesh (icddr,b). This study was approved by the Ethical Review and Research Review Committees of the icddr,b, and the MassGeneral Brigham (MGB) Institutional Review Board, Boston, Massachusetts, USA. Animal work was performed in accordance with all governmental and institutional requirements. All protocols were reviewed and approved by the Massachusetts General Hospital Subcommittee on Research Animal Care (SRAC).

### 3.7 Statistics and graphs

We compared data within groups across time points using Wilcoxon Signed-Rank tests, and across groups using Mann-Whitney U tests. We compared response rates using chi-square (χ^2^) tests. Except for vibriocidal analysis that was one-tailed, all reported P values were two-tailed, with a cutoff of P ≤ 0.05 considered a threshold for statistical significance. We performed statistical analyses using GraphPad Prism 5 (GraphPad Software, Inc.)

## 4 Results

### 4.1 Production and characterization of CCV

**Fig. 2:**
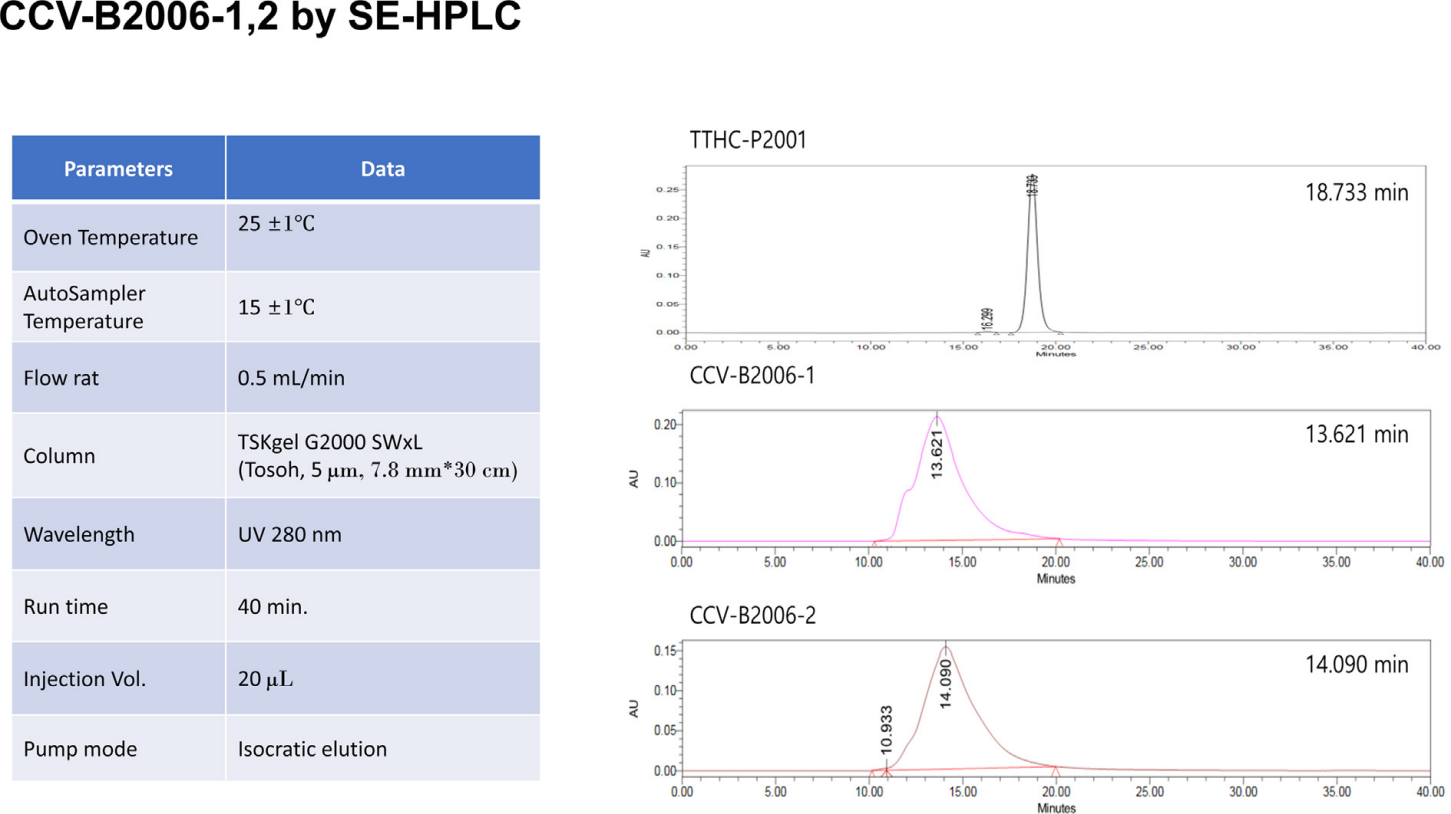
**Analyses of two different batches of CCV using SE-HPLC**. CCVs and rTTHc were analyzed using TSKgel G2000SWxL column on Waters HPLC system at 25 °C, eluted with 10 mM sodium phosphate, 150 mM NaCl, pH 7.2 at 0.5 mL/min flow rate. Comparison of the three analytes shows no unconjugated rTTHc in any of the conjugates. TTHC-P2001: unconjugated rTTHc; CCV-B2006-1 and CCV-B2006-2: Cholera conjugate vaccines containing rTTHc conjugated to OSP (CCV-B2006-1–4.4 OSP:1 rTTHc; CCV-B2006-2 – 3.2 OSP:1 rTTHc).

**Fig. 3:**
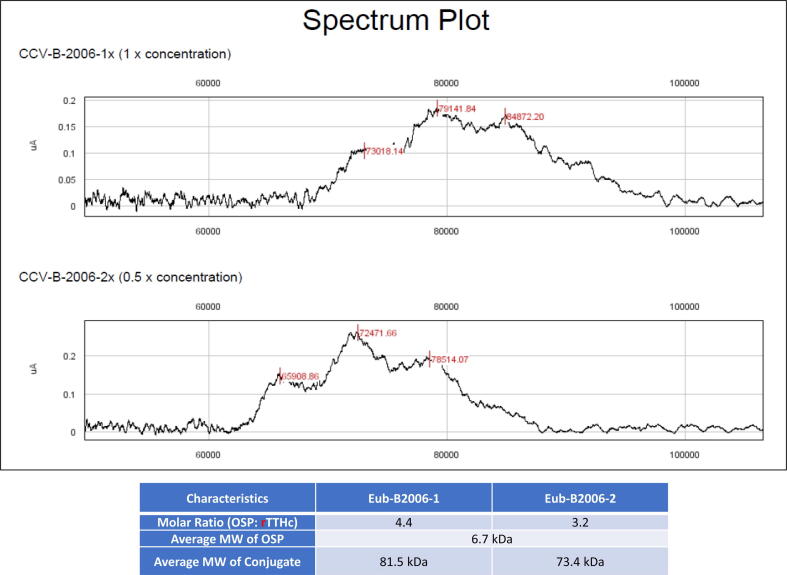
**SELDI-TOF-MS analysis of CCV to determine the average molecular weight (M.w.) of OSP and the molar ratio ofOSP to rTTHc in the conjugates.** The average M.w. of OSP (6.7 kDa) is calculated based on the Mass difference of two neighboring peaks in the SELDI-TOF-MS (Surface-Enhanced Laser Desorption Ionization-Time of Flight-Mass Spectrometry) figures. The average molar ratio (loading) of OSP to rTTHc is determined by the intensity of different peaks. 4.4 is determined to be the loading of Eub-B2006-1 and 3.2 to be the loading of Eub-B2006-2. Average M.w. of conjugate = 52 kDa + 6.7 kDa × loading.

OSP manufacturing processes were successfully optimized and scaled up to 100 L fermentation scale; yield of purified OSP (Supplemental Figs. 1, 2) was ∼ 0.3 g/L. rTTHc manufacturing processes were successfully optimized and scaled up to 50 L fermentation scale; yield of purified rTTHc (Supplemental Fig. 3) was ∼ 1.8 g/L. OSP, rTTHc, and CCV EuB2006-1 and EuB2006-2 were produced and characterized as detailed in Table 1 and Figs. 2, 3.

**Table 1:** Characterization of cholera conjugate vaccine (CCV).

**Process**	**Parameter**	**Eub-B2006-1**	**Eub-B2006-2**
OSP-DSE labelling	OSP, g (%)	3.4 (100%)	
	OSP-DSE, g (%)	3.4 (100%)	
rTTHc concentration	rTTHc, g (%)	2.4 (100%)	
	Concentrated rTTHc, g (%)	1.6 (67%)	
Conjugate reaction	Conjugate volume (mL)	65	130
	OSP:rTTHc, g	1.7:0.8	
	OSP:rTTHc molar ratio	16.5:1	
Final conjugate result	OSP:rTTHc molar ratio	4.4:1	3.2:1
	Combination of OSP & rTTHc, g	1.47	1.95
	OSP overall recovery, g (%)	0.53 (31%)	0.57 (34%)

OSP, O-specific polysaccharide; DSE, dimethyl squarate ester; rTTHc, recombinant tetanus toxoid heavy chain fragment; g, grams

### 4.2 Immunoreactivity of CCV with convalescent human sera

**Fig. 4:**
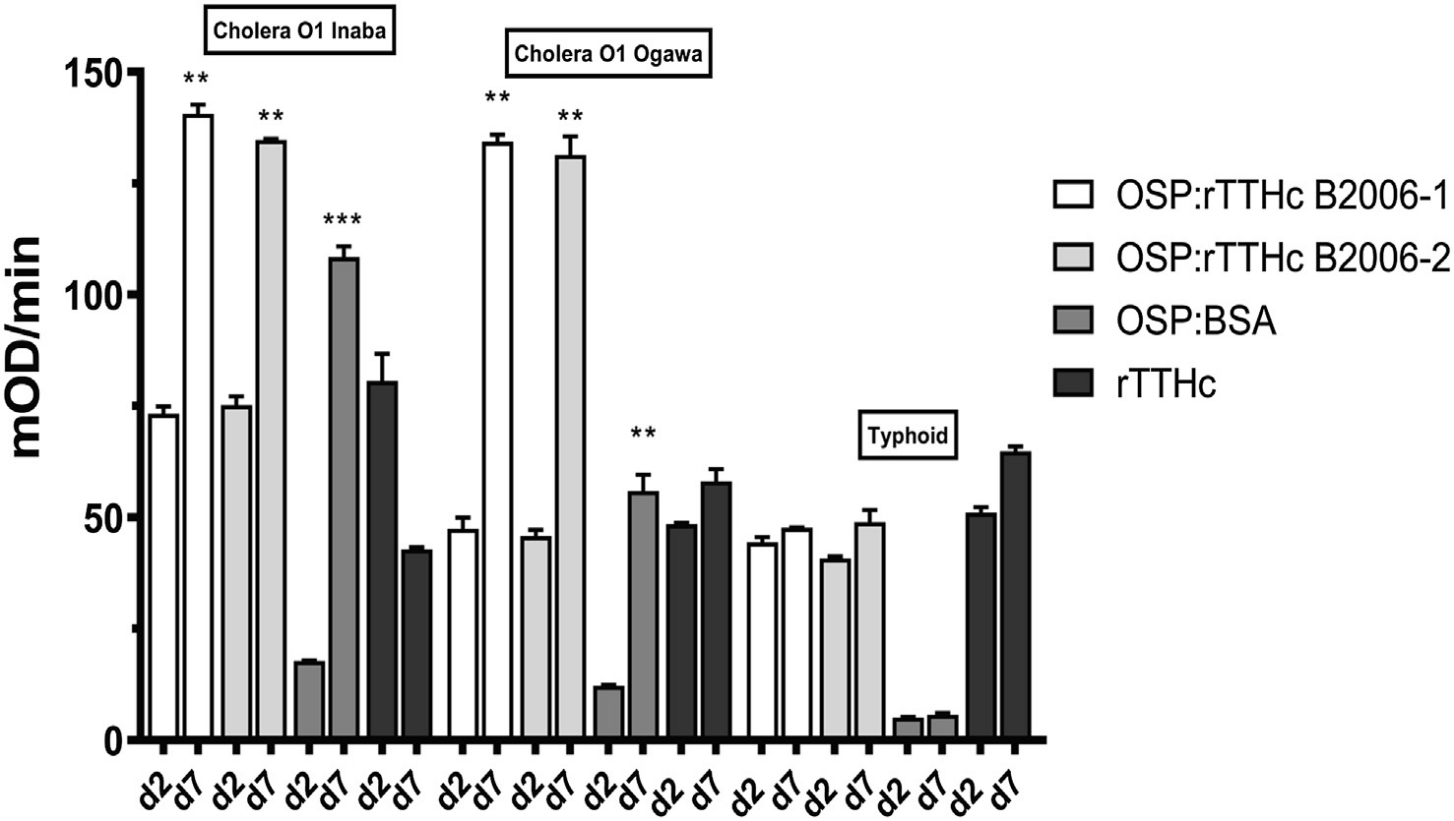
**Immunoreactivity in human plasma of OSP:rTTHc, OSP:BSA, and rTTHc.** Immunoreactivity of two production batches of OSP:rTTHc, OSP:BSA and rTTHc was measured in day 2 versus day 7 plasma of patients with cholera caused by *V. cholerae* O1 serotype Inaba, cholera caused by *V. cholerae* O1 serotype Ogawa, or typhoid fever in Dhaka, Bangladesh. ** denotes a statistically significant increase (P < 0.01) on day 7 compared to day 2; *** p ≤ 0.001.

CCV was recognized by convalescent sera samples from humans recovering from cholera in Bangladesh, and not recognized by patients recovering from typhoid (Fig. 4). In the cholera-related analysis, immunoreactivity was comparable using plasma from humans recovered by cholera caused by Inaba and Ogawa serotypes (Fig. 4).

### 4.3 Immunogenicity of CCV in preclinical analysis

**Fig. 5:**
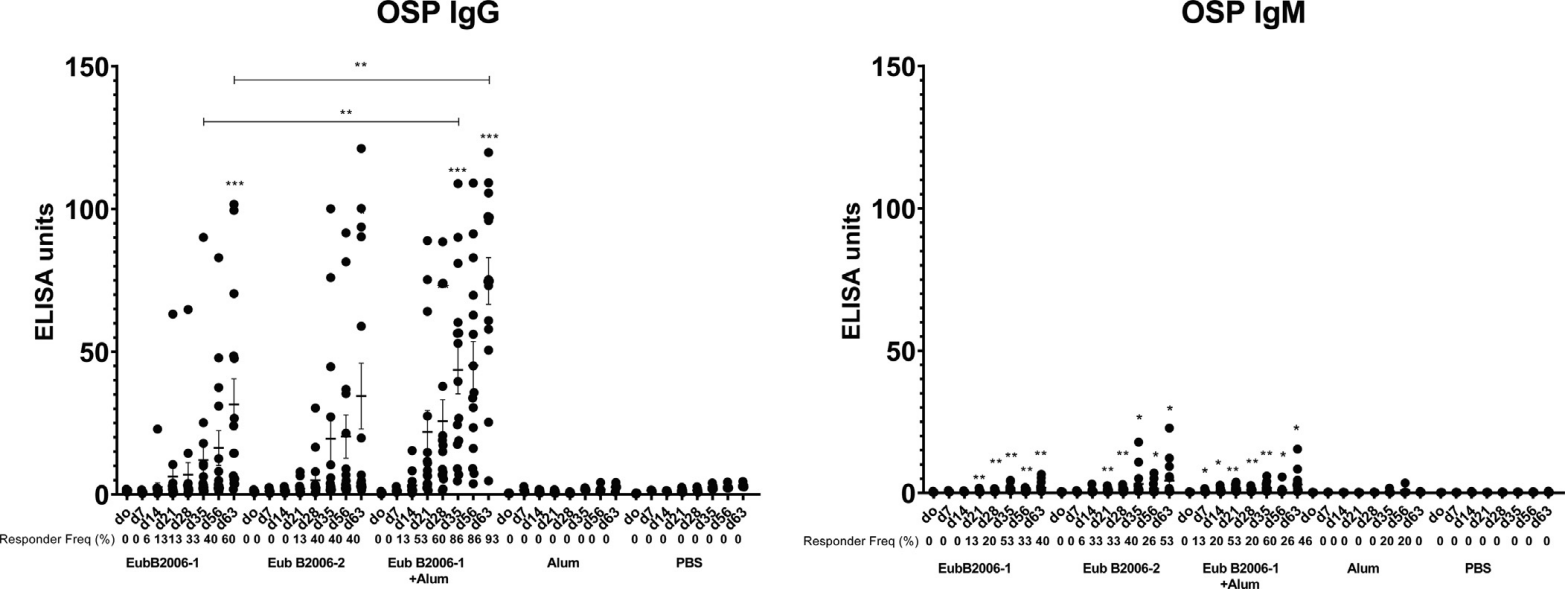
Serum IgG (A) and IgM (B) responses at different time points against O-specific polysaccharide (OSP) in various cohorts of mice following intramuscular (IM) immunization with various batches of vaccine, with or without adjuvant alum. Dots represent responses in individual mice. Mean and standard error of the mean are reported for each group. * denotes a statistically significant difference (P < 0.05) from baseline (day 0) response; ** p ≤ 0.01; *** p ≤ 0.001. Responder frequencies are also listed. Statistically significant differences among compared cohorts are also represented. See Supplemental Material for responder frequency comparisons.

**Fig. 6:**
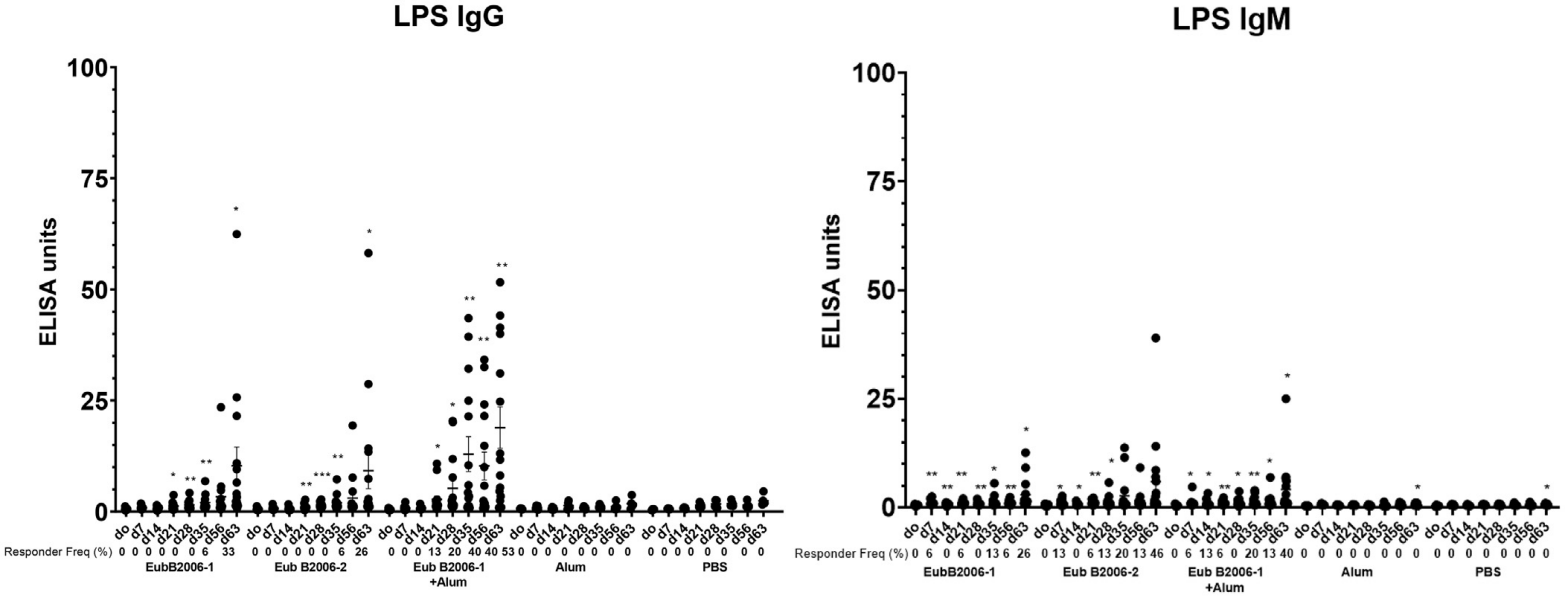
Serum IgG (A) and IgM (B) responses at different time points against lipopolysaccharide (LPS) in various cohorts of mice following intramuscular (IM) immunization with various batches of vaccine, with or without adjuvant alum. Dots represent responses in individual mice. Mean and standard error of the mean are reported for each group. * denotes a statistically significant difference (P < 0.05) from baseline (day 0) response; ** p ≤ 0.01; *** p ≤ 0.001. Responder frequencies are also listed. See Supplemental Material for responder frequency comparisons.

**Fig. 7:**
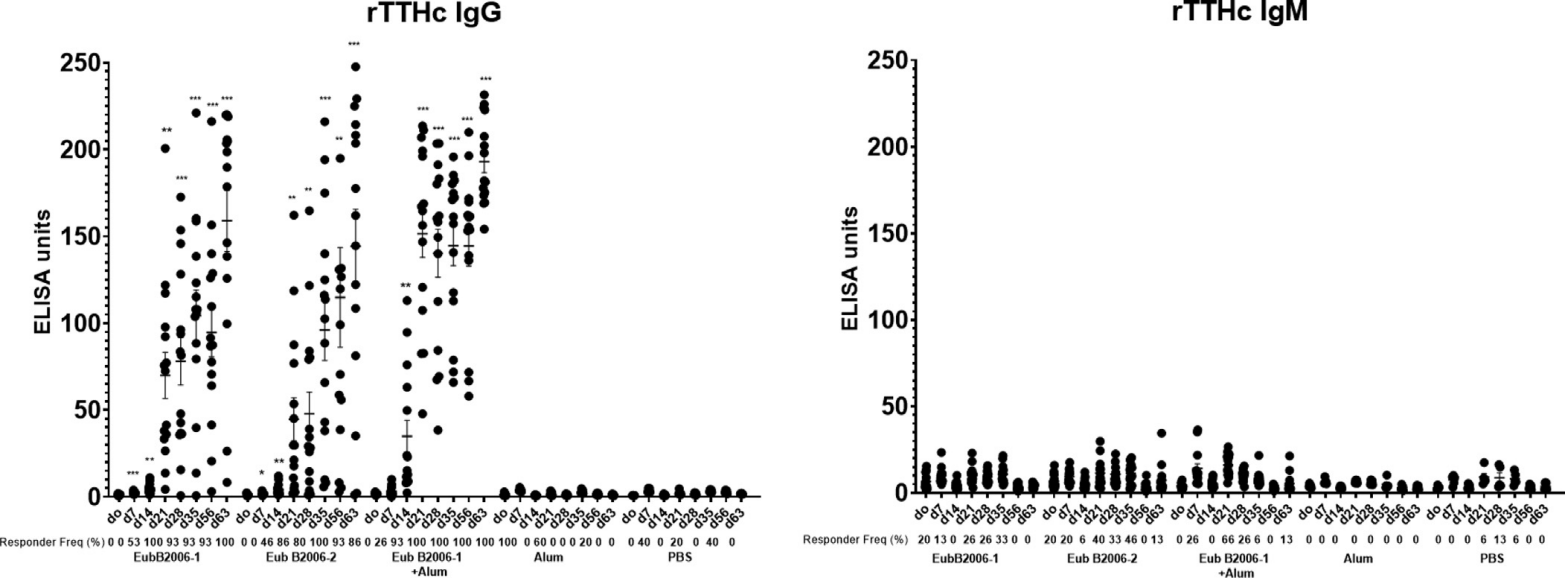
Serum IgG (A) and IgM (B) responses at different time points against rTTHc in various cohorts of mice following intramuscular (IM) immunization with various batches of vaccine, with or without adjuvant alum. Dots represent responses in individual mice. Mean and standard error of the mean are reported for each group. * denotes a statistically significant difference (P < 0.05) from baseline (day 0) response; ** p ≤ 0.01; *** p ≤ 0.001. Responder frequencies are also listed. See Supplemental Material for responder frequency comparisons.

**Fig. 8:**
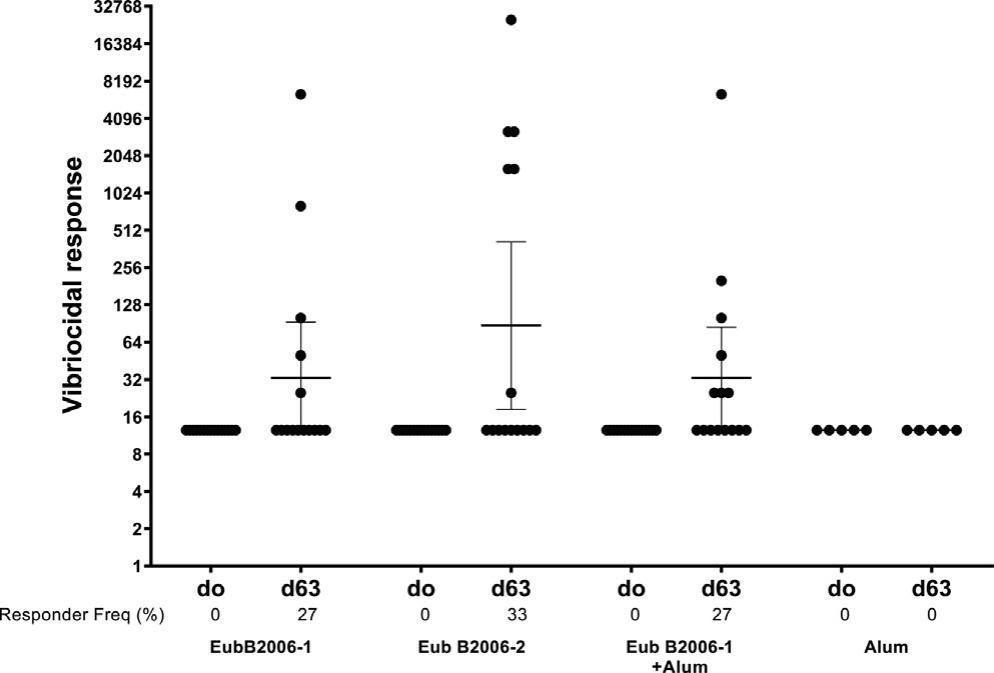
**Vibriocidal responses in vaccinated cohorts of mice**. Dots represent responses in individual mice on day 0 (d0) and day 63 (d63). Mean and standard error of the mean are reported for each group. See Supplemental Material for responder frequency comparisons.

**Fig. 9:**
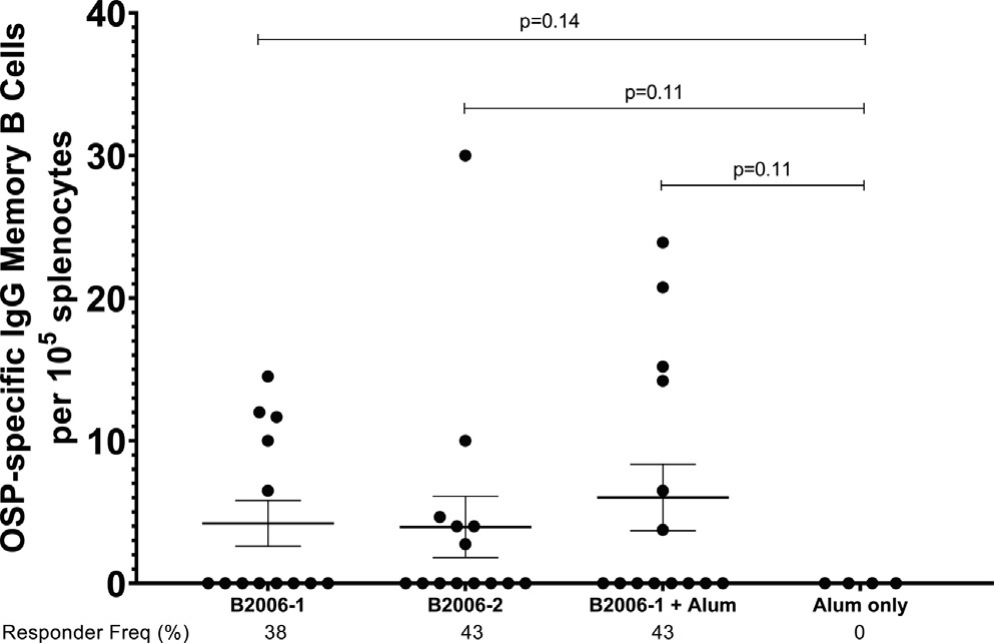
**Memory B cell IgG responses in spleen targeting O-specific polysaccharide (OSP) in various cohorts of mice receiving CCV with or without adjuvantative alum.** Dots represent responses in individual mice on day 0 (d0) and day 63 (d63). Mean and standard error of the mean are reported for each group. See Supplemental Material for responder frequency comparisons.

Mice immunized with CCV developed OSP-specific IgG (Fig. 5A; p < 0.001). Immune responses were comparable following immunization with two distinct production batches of CCV (EubB2006-1 and EubB2006-2). Immune responses were increased in the presence of alum (Fig. 5A; p < 0.01). Immune responses were detected following a single immunization, were significantly elevated 7 days following dose 2 in the adjuvanted cohort (p < 0.05) and increased further following a day 56 booster (p < 0.05). OSP-specific IgM responses were also detected following vaccination with CCV and were significantly elevated 7 days following a single dose in the adjuvanted group (Fig. 5B; p < 0.05). LPS-specific IgG and IgM responses were detected following vaccination with both batches of CCV (Fig. 6). LPS-specific IgM responses were significantly elevated within 7 days of a single vaccination in all cohorts (Fig. 6B; p < 0.05), IgG responses were significantly elevated by day 21 in all cohorts (Fig. 6A; p < 0.05) and continued to increase until sacrifice (p < 0.01). Immunization with CCV also induced rTTHc-specific IgG responses (Fig. 7A; P < 0.001), but not rTTHc-specific IgM responses (Fig. 7B). Vaccination with CCV induced vibriocidal responses; these responses did not increase in the presence of the alum adjuvant (Fig. 8). OSP-specific memory B cell IgG responses were also detected in animals vaccinated with CCV with and without alum, but not detected in animals vaccinated with alum alone (Fig. 9). In the cholera challenge model, we found a significant increase in survival between mice challenged with wild type *V. cholerae* O1 Inaba N16961 mixed with sera collected from mice immunized with CCV (81% survival at 36 h), compared to mice challenged using sera from mice vaccinated with placebo alone (46% survival at 36 h; vaccine efficacy 65%; p = 0.04, Table 2; Supplemental Fig. 4). Sera from mice vaccinated with CCV had higher OSP-specific IgG than mice immunized with PBS (Supplemental Fig. 5; p < 0.001). The addition of alum did not further increase survival.

**Table 2:** Survival at 36 h in mice challenged with virulent *V. cholerae* O1 N16961.

**Vaccine cohort**	**N**	**Survival at 36 h**	**Vaccine Efficacy (%)**	**P value**
Placebo-PBS	15	46%	–	–
Eub-B2006-1	16	81%	65%	**0.04**
Eub-B2006-1/alum	11	73%	50%	0.18

## 5 Discussion

Here, we describe scalable production of a cholera conjugate vaccine (CCV), evaluation of immunoreactivity using serum derived from humans surviving cholera, and preclinical assessment of CCV. Currently available oral cholera vaccines (OCVs) have been a transformational addition to global cholera control programs; however, a *meta*-analysis suggests a two dose efficacy of 58% overall for OCVs, with 64% in individuals older than 5 years of age, and only 30% in children younger than 5 years of age [35]. With regard to duration, a *meta*-analysis generated average estimates of OCV efficacy approximating 55–60% in the first 2 years following vaccination, falling to 39% and 26% in years 3 and 4 post-vaccination, respectively [35]. The lower efficacy in children under 5 years has limited the utility of incorporating OCVs into EPI schedules, despite the fact that children are at particular risk of cholera in endemic areas. In such areas, children younger than 5 years of age have a 2–4 times higher incidence rate of cholera than those in the general population [3], [10], [11]. Next generation cholera vaccines that induce high-level and durable protective immunity in young children and that can be incorporated into EPI schedules could further transform global cholera control efforts.

Historically, the vibriocidal response has been used as an imperfect correlate of protection against cholera. Although there is a correlation of vibriocidal titers and protection against cholera, there is no absolute vibriocidal value that predicts protection against cholera [36], and individuals can be protected against cholera with no increase of vibriocidal antibody response following exposure and subsequent challenge, suggesting that other immune responses actually mediate protection against cholera [37]. The vibriocidal response is an *in vitro* bactericidal assay largely comprised of IgM and IgG responses against *V. cholerae* OSP [38], with bacteriocidal activity being cell-free and complement-dependent, resting on the formation in bacterial membranes of a membrane attack complex (MAC) comprised of the terminal components of complement. The shortcomings of this assay for judging protection against the non-invasive mucosal *V. cholerae* pathogen should be considered in the context that there is no convincing evidence that the terminal complement components required to form a membrane attack complex are present within the intestinal lumen in the setting of an intact intestinal epithelium, the fact that IgA does not bind complement via the classical pathway (despite IgA being the primary antibody expressed at the mucosal surface and secreted into the intestinal lumen), and that data do not suggest any evidence for actual killing of *V. cholerae* within the intestines of infected humans or animals who are protected from disease [39], [40], [41].

In summary, there is no evidence that bacteriocidal activity is a mechanism of protection against cholera. In comparison, a growing body of evidence suggests that protection against cholera is mediated by OSP-specific antibody responses [41]. The vibriocidal response largely targets the O-specific polysaccharide (OSP) of *V. cholerae* [38]. OSP-specific antibody and memory B cell responses correlate with protection against cholera in household contacts of cholera index patients in Bangladesh [16]; and in North American recipients of an oral cholera vaccine, OSP-specific antibody responses correlate with protection against cholera in experimental challenge studies [15]. OSP-specific antibody responses inhibit *V. cholerae* motility, and this effect may be mechanistically involved in protection against cholera within the intestinal lumen of infected humans [41]. Blocking the ability of the highly mobile and freely-swimming *V. cholerae* pathogens from reaching their target ecological niche within the intestinal lumen of infected humans and delivering cholera toxin to intestinal epithelial cells may be protective [41], [42].

Over 200 serogroups of *V. cholerae* exist, with serogroups O1 and O139 capable of causing epidemic cholera, and protection against cholera is serogroup-specific [43], [44], [45]. Cholera caused by O139 has disappeared as a clinically significant cause of cholera for unclear reasons. The OSP of *V. cholerae* is a relatively simple structure, comprised of repetitive (1 → 2)α-linked D-perosamines, which are N-acylated with 3-deoxy-L-*glycero*-tetronic acid, with a chain length of approximately 7–22 perosaminyl residues. *V. cholerae* O1 organisms can be classified into two major serotypes, Ogawa and Inaba, based on the presence or absence, respectively, of a methyl group on the upstream terminal 2-OH moiety of OSP [46]. OSP is attached via a core oligosaccharide to lipid A to comprise the *V. cholerae* LPS. Previous infection with Ogawa provides protection against subsequent Ogawa infection, while previous infection with Inaba provides more complete protection against both subsequent Inaba and Ogawa infection [47], [48], [49]. For these reasons, we selected Inaba OSP as the pertinent immunogen for CCV, and use *V. cholerae* O1 Inaba El Tor PIC018, a well-characterized strain isolated in 2007 from a patient in Bangladesh, as our source strain (GENBANK: https://www.ncbi.nlm.nih.gov/nuccore/NZ_LQHM00000000.1).

Our production protocol uses mild hydrolysis of LPS from *V. cholerae* directly in biofermenters, retaining intact the OSP-core component, which contains an active glucosamine group. We then use squarate chemistry to directly link *V. cholerae* OSP via core oligosaccharide to carrier protein. This approach does not require introduction of an unrelated linker, minimizes derivatization steps, simplifies manufacturing, and significantly lowers the cost of production of CCV. It results in conjugates that are not cross-linked, easy to characterize, and display OSP in a sun-burst, single-point-attachment in a manner that recapitulates how OSP is presented on *V. cholerae*. It should be noted that other Gram-negative bacterial pathogens also contain a single active glucosamine in core oligosaccharide, including *Shigella* spp., suggesting that our conjugation approach could have platform utility in producing multi-valent enteric vaccines.

We chose a recombinant 52 kDa fragment of tetanus toxoid heavy chain as carrier protein. This protein was optimized for high level expression and purification in *E. coli* for production purposes, and functions very well in conjugation reactions and as a carrier protein [30], [50], [51]. We avoided using a diphtheria toxoid derivative, with a goal of minimizing exposure to a common carrier protein that could result in group blunting [52], while identifying rTTHc as a novel carrier protein that could have platform functionality in multivalent vaccines. The CCV used in this analysis contained approximately 3–4.5 mol of OSP per mole of rTTHc. We previously analyzed various molar loading of OSP to carrier protein and found that a molar loading of 3–5 was optimal [30]. We also previously analyzed various vaccination doses of CCV (10–50 µg of OSP per dose) [30] in preclinical immunogenicity studies, and found comparable immune responses across the range of doses; we therefore focused our current pre-clinical analysis using a dose of 10 µg of saccharide. In our current analysis, we assessed two distinct production batches of CCV, with OSP to rTTHc molar loading differing by ∼ 1.4 fold (4.4:1 versus 3.2:1) We found the two batches comparable in their immunoreactivity and immunogenicity, suggesting that process parameters for conjugate drug substance manufacturing can be set widely enough to ensure easy operation and scale-up. Our production approach resulted in an OSP-conjugation efficiency of 30–35%, which is higher than that of other conjugation technologies and, in addition, our approach obviates the need to introduce linkers/spacers between OSP and carrier protein – significantly simplifying manufacturing processes. Subsequent engineering and cGMP production of CCV have shown comparable reproducibility and efficiency to those reported here, suggesting a robust production protocol. Assuming a human CCV dose of approximately 10 µg of polysaccharide, our results suggest that over 300,000 doses of CCV could be produced per 10 g of input-OSP.

We found that OSP displayed on CCV was recognized by plasma from humans recovering from cholera caused by both of the major serotypes of *V. cholerae* O1: Inaba and Ogawa. This is not unexpected since the two serotypes have high homology, differing only in the presence or absence of a methyl group on the terminal saccharide of OSP. Immune responses and protection against cholera is not serotype specific [47], [48], [49], and our results suggest that CCV results in similar cross-serotype immunity and displays OSP in a immunologically relevant manner.

In our preclinical immunogenicity analysis, we found that OSP-specific immune responses were boosted in the presence of aluminum adjuvant. The presence of alum did not markedly increase survival in our protection assay. There was a trend in increase in LPS-specific and rTTHc-specific IgG but not IgM responses in the presence of alum. There was, however, no evident increase in vibriocidal responses, perhaps reflecting the absence of boosting of IgM responses by alum. We did find that vaccination with CCV with or without alum induced IgM responses in addition to IgG responses but did not induce rTTHc-specific IgM responses. This may reflect different immune processing of polysaccharide versus protein antigen. Analysis of CCV showed absence of unconjugated OSP. Interestingly, vaccination with a typhoid conjugate vaccine similarly induced polysaccharide-specific IgM and IgA responses not only in humans vaccinated in a typhoid endemic zone (with possible boosting of pre-existing responses) [53], but also, quite strikingly, in humans with no previous exposure to *Salmonella* Typhi in the United Kingdom [54]. In these immunologically naïve individuals, parenteral vaccination with typhoid conjugate also induced mucosally-homing immune responses [54]. These data, and the fact that parenteral injection of a previous cholera vaccine induced 40–70% protection against cholera [23], [24], [25], [26], [27], [28], [29], strongly suggest that parenteral injection of polysaccharides, including in conjugate form, can induce mucosal responses.

Analysis of previous conjugate vaccines are also consistent with induction of impactful mucosal responses. It has long been recognized that parenteral immunization can boost both mucosal and systemic immune responses that protect against cholera in humans residing in cholera-endemic areas, possibly through boosting of pre-existing immune responses, including IgM and secretory IgA in intestinal secretions, milk and saliva [55], [56], [57], [58]. Parenterally administered conjugate vaccines have also been found to be highly effective against many mucosal pathogens, including *Haemophilus influenzae* b, *Streptococcus pneumoniae*, and *Neissseria meningitidis*, not only in preventing invasive disease but also in decreasing mucosal colonization and carriage with serotypes included in the conjugate vaccine, suggesting a mucosal effector function [59], [60], [61], [62], [63], [64]. These effects on carriage were not seen when vaccines containing only unconjugated polysaccharide were used [65], [66].

IgA and IgM are actively secreted into the lumen at the intestinal surface, but anti-polysaccharide IgG is also detected in mucosal fluids following parenteral vaccination with conjugate vaccines, even in young infants, but not following vaccination with unconjugated polysaccharide [67], [68], [69], [70], [71], [72]. These mucosal IgG responses correlate with serum IgG responses much more closely than mucosal IgA responses correlate with serum IgA responses, suggesting local mucosal production of IgA, but transudation or exudation of serum IgG into mucosal fluids following parenteral vaccination with polysaccharide-conjugate vaccines [67], [68], [69], [70], [71], [72]. Exudation transfer of serum antibodies including IgG may be especially prominent in communities at risk not just of cholera but of tropical-environmental enteropathy and mucosal breakdown with lack of intestinal epithelial integrity [73]. IgG in mucosal fluids induced following vaccination with a polysaccharide-conjugate vaccine may also have distinct anti-bacterial effects compared to those induced by vaccination with the same unconjugated polysaccharides [74].

In conclusion, we report here development and scalable production of a cholera conjugate vaccine. We demonstrate that the vaccine displays *V. cholerae* OSP in an immunologically relevant manner, is recognized by convalescent phase sera from both Inaba and Ogawa serotype-associated cholera, and induces protective and durable memory immune responses in pre-clinical evaluation studies. OSP is the target antigen for immune responses that mediate protection against cholera, and polysaccharide-specific immune responses following immunization with conjugate vaccines can be protective at mucosal surfaces. Our approach is an improvement over previous unconjugated parenteral cholera vaccines that were as protective as oral cholera vaccines, but that induced only short-term protection requiring frequent re-vaccination. Our approach also addresses the shortcomings of currently available oral cholera vaccines that suffer from poor protective efficacy in children under 5 years of age, induction of relatively short-term protection, and difficulties in incorporating into EPI schedules. Our data suggest that CCV, following completion of a GLP toxicology study, should be evaluated in a phase 1 trial in humans to evaluate safety and immunogenicity. This and subsequent evaluations should include assessment of immune responses associated with protection against cholera, including OSP-LPS-specific responses in serum, OSP-LPS-specific responses in mucosal samples (antibody secretory cell analysis with mucosal homing markers), and OSP-LPS memory B cell responses.

## Declaration of Competing Interest

The authors declare that they have no known competing financial interests or personal relationships that could have appeared to influence the work reported in this paper.

## Appendix A Supplementary material

Figure S1 ^1^H-NMR comparison of OSP produced by Eubiologics (black) with standard (red) [75]. ^1^H-NMR experiments were measured in D 2 O at 25 ° C in Shigemi NMR tube, at 600 MHz on a Bruker Avance Spectrometer with a TCI cryoprobe. Samples were prepared at ~10 mg/mL concentration.

Figure S2. ^13^C-NMR Comparison of OSP produced by Eubiologics (black) with standard (red) [75]. ^13^C-NMR experiments were measured in D 2 O at 25 ° C in Shigemi NMR tube, at 150 MHz on a Bruker Avance Spectrometer with a TCI cryoprobe. Samples were prepared at ~10 mg/mL concentration.

Figure S3 Purified rTTHc analyzed by SE-HPLC compared with standard . rTTHc was analyzed using TSKgel G2000SWxL column on Waters HPLC system at 25° C, eluted with 10 mM sodium phosphate, 150 mM NaCl, pH 7.2) at 0.5 mL/min flow rate.

Figure S4 Survival curves of experimentally challenged animals by cohort. Three-to-five day old CD-1 mouse pups were orally gavaged with 50 μl of a preparation containing 1x10^9^ CFU of wild type *V. cholerae* N16961 mixed with a 1:1 dilution of pooled day 63 serum from mice intramuscularly immunized with phosphate buffered saline (PBS) or conjugate vaccine ( OSP:rTTHc ) with or without immunoadjuvantative alum.

Figure S5 OSP-specific IgG responses in pooled day 63 sera used in animal challenge studies by vaccine cohort . See text for cohorts. *** P<0.001.

## References

[1] Harris JB, LaRocque RC, Qadri F, Ryan ET, Calderwood SB. Cholera. Lancet. 2012;379(9835):2466-76. PMC3761070. 10.1016/S0140-6736(12)60436-X PMC3761070 22748592

[2] Ali M, Nelson AR, Lopez AL, Sack DA. Updated global burden of cholera in endemic countries. PLoS Negl Trop Dis. 2015;9(6):e0003832. PMC4455997. 10.1371/journal.pntd.0003832 PMC4455997 26043000

[3] Pezzoli L, Oral Cholera Vaccine Working Group of the Global Task Force on Cholera C. Global oral cholera vaccine use, 2013-2018. Vaccine. 2020;38 Suppl 1:A132-A40. PMID: 31519444. 10.1016/j.vaccine.2019.08.086 PMC10967685 31519444

[4] Legros D, Partners of the Global Task Force on Cholera Control. Global Cholera Epidemiology: opportunities to reduce the burden of cholera by 2030. J Infect Dis. 2018;218(suppl_3):S137-S40. PMC6207143. 10.1093/infdis/jiy486 PMC6207143 30184102

[5] Sur D, Kanungo S, Sah B, Manna B, Ali M, Paisley AM, et al. Efficacy of a low-cost, inactivated whole-cell oral cholera vaccine: results from 3 years of follow-up of a randomized, controlled trial. PLoS Negl Trop Dis. 2011;5(10):e1289. PMC3196468. 10.1371/journal.pntd.0001289 PMC3196468 22028938

[6] Sinclair D, Abba K, Zaman K, Qadri F, Graves PM. Oral vaccines for preventing cholera. Cochrane Database Syst Rev. 2011;(3):CD008603. PMC6532691. 10.1002/14651858.CD008603.pub2 PMC6532691 21412922

[7] Leung D.T., Rahman M.A., Mohasin M., Patel S.M., Aktar A., Khanam F. (2012). Memory B cell and other immune responses in children receiving two doses of an oral killed cholera vaccine compared to responses following natural cholera infection in Bangladesh. Clin Vaccine Immunol.

[8] Kanungo S., Paisley A., Lopez A.L., Bhattacharya M., Manna B., Kim D.R. (2009). Immune responses following one and two doses of the reformulated, bivalent, killed, whole-cell, oral cholera vaccine among adults and children in Kolkata, India: a randomized, placebo-controlled trial. Vaccine..

[9] Saha A., Chowdhury M.I., Khanam F., Bhuiyan M.S., Chowdhury F., Khan A.I. (2011). Safety and immunogenicity study of a killed bivalent (O1 and O139) whole-cell oral cholera vaccine Shanchol, in Bangladeshi adults and children as young as 1 year of age. Vaccine..

[10] Alberti K.P., Guthmann J.P., Fermon F., Nargaye K.D., Grais R.F. (2008). Use of Lot Quality Assurance Sampling (LQAS) to estimate vaccination coverage helps guide future vaccination efforts. Trans R Soc Trop Med Hyg..

[11] Ali M., Lopez A.L., Ae You Y., Eun Kim Y., Sah B., Maskery B. (2012). The global burden of cholera. Bull World Health Organ..

[12] Gavi-the Vaccine Alliance; Cholera Supply and Procurement Roadmap UPDATE 14th December 2018; p4 https://www.gavi.org/our-alliance/market-shaping/supply-and-procurement-roadmaps2018 [June 3, 2021].

[13] Global Task Force on Cholera Control GTFCC: Cholera Vaccines Research Priorities; OCV Working Group 2019; p3 https://www.gtfcc.org/wp-content/uploads/2020/08/6th-gtfcc-working-group-on-ocv-meeting-2019-research-priorities.pdf2019 [June 3, 2021].

[14] Global Task Force on Cholera Control GTFCC: Cholera Roadmap Research Agenda; 2021Vaccines Research Priorities; p16 https://www.gtfcc.org/wp-content/uploads/2021/01/gtfcc-global-roadmap-research-agenda-full-report-with-methodology.pdf2021 [June 3, 2021].

[15] Islam K, Hossain M, Kelly M, Mayo Smith LM, Charles RC, Bhuiyan TR, et al. Anti-O-specific polysaccharide (OSP) immune responses following vaccination with oral cholera vaccine CVD 103-HgR correlate with protection against cholera after infection with wild-type *Vibrio cholerae* O1 El Tor Inaba in North American volunteers. PLoS Negl Trop Dis. 2018;12(4):e0006376. PMC5906022. 10.1371/journal.pntd.0006376 PMC5906022 29624592

[16] Aktar A, Rahman MA, Afrin S, Akter A, Uddin T, Yasmin T (2018). Plasma and memory B cell responses targeting O-specific polysaccharide (OSP) are associated with protection against *Vibrio cholerae* O1 infection among household contacts of cholera patients in Bangladesh. PLoS Negl Trop Dis.

[17] MacDonald N.E., Halperin S.A., Law B.J., Forrest B., Danzig L.E., Granoff D.M. (1998). Induction of immunologic memory by conjugated vs plain meningococcal C polysaccharide vaccine in toddlers: a randomized controlled trial. JAMA.

[18] Pollard A.J., Perrett K.P., Beverley P.C. (2009). Maintaining protection against invasive bacteria with protein-polysaccharide conjugate vaccines. Nat Rev Immunol..

[19] Clutterbuck EA, Lazarus R, Yu LM, Bowman J, Bateman EA, Diggle L, et al. Pneumococcal conjugate and plain polysaccharide vaccines have divergent effects on antigen-specific B cells. J Infect Dis. 2012;205(9):1408-16. PMC3324398. 10.1093/infdis/jis212 PMC3324398 22457293

[20] Ochiai R.L., Khan M.I., Soofi S.B., Sur D., Kanungo S., You Y.A. (2014). Immune responses to Vi capsular polysaccharide typhoid vaccine in children 2 to 16 years old in Karachi, Pakistan, and Kolkata, India. Clin Vaccine Immunol.

[21] Thiem V.D., Lin F.-Y., Canh D.G., Son N.H., Anh D.D., Mao N.D. (2011). The Vi conjugate typhoid vaccine is safe, elicits protective levels of IgG anti-Vi, and is compatible with routine infant vaccines. Clin Vaccine Immunol.

[22] Truck J, Mitchell R, Jawad S, Clutterbuck EA, Snape MD, Kelly DF, et al. Divergent memory B cell responses in a mixed infantpneumococcal conjugate vaccine schedule. Pediatr Infect Dis J. 2017;36(5):e130–e5. PMID: 28027283. 10.1097/INF.0000000000001497 28027283

[23] Benenson AS, Mosley WH, Fahimuddin M, Oseasohn RO. Cholera vaccine field trials in east Pakistan. 2. Effectiveness in the field. Bull World Health Organ. 1968;38(3):359–72. PMC2554494. PMC2554494 5302329

[24] Benenson AS, Joseph PR, Oseasohn RO. Cholera vaccine field trials in east Pakistan. 1. Reaction and antigenicity studies. Bull World Health Organ. 1968;38(3):347–57. PMC2554479. PMC2554479 5302328

[25] Das Gupta A, Sinha R, Shrivastava DL, De SP, Taneja BL, Rao MS, et al. Controlled field trial of the effectiveness of cholera and cholera El Tor vaccines in Calcutta. Bull World Health Organ. 1967;37(3):371–85. PMC2554277. PMC2554277 5301381

[26] Azurin JC, Cruz A, Pesigan TP, Alvero M, Camena T, Suplido R, et al. A controlled field trial of the effectiveness of cholera and cholera El Tor vaccines in the Philippines. Bull World Health Organ. 1967;37(5):703-27. PMC2554919. PMC2554919 5300874

[27] Mosley WH, Benenson AS, Barui R. A serological survey for cholera antibodies in rural east Pakistan. 2. A comparison of antibody titres in the innunized and control populationd of a cholera-vaccine field-trial area and the relation of antibody titre to cholera case rate. Bull World Health Organ. 1968;38(3):335–46. PMC2554488. PMC2554488 5302923

[28] A controlled field trial of the effectiveness of various doses of cholera El Tor vaccine in the Philippines. Bull World Health Organ. 1968;38(6):917-23. PMC2554519. PMC2554519 5303665

[29] Mosley WH, McCormack WM, Fahimuddin M, Aziz KM, Rahman AS, Chowdhury AK, et al. Report of the 1966-67 cholera vaccine field trial in rural East Pakistan. I. Study design and results of the first year of observation. Bull World Health Organ. 1969;40(2):177–85. PMC2554610. PMC2554610 5306538

[30] Sayeed MA, Bufano MK, Xu P, Eckhoff G, Charles RC, Alam MM, et al. A Cholera Conjugate vaccine containing O-specific polysaccharide (OSP) of *V. cholerae* O1 Inaba and recombinant fragment of tetanus toxin heavy chain (OSP:rTTHc) induces serum, memory and lamina proprial responses against OSP and is protective in mice. PLoS Negl Trop Dis. 2015;9(7):e0003881. PMC4495926. 10.1371/journal.pntd.0003881 PMC4495926 26154421

[31] Alam MM, Bufano MK, Xu P, Kalsy A, Yu Y, Freeman YW, et al. Evaluation in mice of a conjugate vaccine for cholera made from *Vibrio cholerae* O1 (Ogawa) O-specific polysaccharide. PLoS Negl Trop Dis. 2014;8(2):e2683. PMC3916310. 10.1371/journal.pntd.0002683 PMC3916310 24516685

[32] Tarique A.A., Kalsy A., Arifuzzaman M., Rollins S.M., Charles R.C., Leung D.T. (2012). Transcutaneous immunization with a *Vibrio cholerae* O1 Ogawa synthetic hexasaccharide conjugate following oral whole-cell cholera vaccination boosts vibriocidal responses and induces protective immunity in mice. Clin Vaccine Immunol.

[33] Rollenhagen J.E., Kalsy A., Saksena R., Sheikh A., Alam M.M., Qadri F. (2009). Transcutaneous immunization with a synthetic hexasaccharide-protein conjugate induces anti-*Vibrio cholerae* lipopolysaccharide responses in mice. Vaccine..

[34] Slifka M.K., Ahmed R. (1996). Limiting dilution analysis of virus-specific memory B cells by an ELISPOT assay. J Immunol Methods.

[35] Bi Q., Ferreras E., Pezzoli L., Legros D., Ivers L.C., Date K. (2017). Protection against cholera from killed whole-cell oral cholera vaccines: a systematic review and meta-analysis. Lancet Infect Dis.

[36] Harris J.B., LaRocque R.C., Chowdhury F., Khan A.I., Logvinenko T., Faruque A.S.G. (2008). Susceptibility to *Vibrio cholerae* infection in a cohort of household contacts of patients with cholera in Bangladesh. PLoS Negl Trop Dis..

[37] Clements M.L., Levine M.M., Young C.R., Black R.E., Lim Y.L., Robins-Browne R.M. (1982). Magnitude, kinetics, and duration of vibriocidal antibody responses in North Americans after ingestion of *Vibrio cholerae*. J Infect Dis.

[38] Johnson R.A., Uddin T., Aktar A., Mohasin M., Alam M.M., Chowdhury F. (2012). Comparison of immune responses to the O-specific polysaccharide and lipopolysaccharide of *Vibrio cholerae* O1 in Bangladeshi adult patients with cholera. Clin Vaccine Immunol.

[39] Wang Z., Lazinski D.W., Camilli A., Pirofski L.-A. (2017). Immunity provided by an outer membrane vesicle cholera vaccine Is due to O-antigen-specific antibodies inhibiting bacterial motility. Infect Immun.

[40] Bishop A.L., Schild S., Patimalla B., Klein B., Camilli A. (2010). Mucosal immunization with *Vibrio cholerae* outer membrane vesicles provides maternal protection mediated by antilipopolysaccharide antibodies that inhibit bacterial motility. Infect Immun.

[41] Charles R.C., Kelly M., Tam J.M., Akter A., Hossain M., Islam K. (2020). Humans surviving cholera develop antibodies against *Vibrio cholerae* O-specific polysaccharide that inhibit pathogen motility. mBio..

[42] Kauffman R.C., Adekunle O., Yu H., Cho A., Nyhoff L.E., Kelly M. (2021). Impact of immunoglobulin isotype and epitope on the functional properties of *Vibrio cholerae* O-specific polysaccharide-specific monoclonal antibodies. mBio..

[43] Albert M.J., Alam K., Ansaruzzaman M., Qadri F., Sack R.B. (1994). Lack of cross-protection against diarrhea due to *Vibrio cholerae* O139 (Bengal strain) after oral immunization of rabbits with *V. cholerae* O1 vaccine strain CVD103-HgR. J Infect Dis.

[44] Qadri F., Wennerås C., Albert M.J., Hossain J., Mannoor K., Begum Y.A. (1997). Comparison of immune responses in patients infected with *Vibrio cholerae* O139 and O1. Infect Immun.

[45] Waldor M.K., Colwell R., Mekalanos J.J. (1994). The *Vibrio cholerae* O139 serogroup antigen includes an O-antigen capsule and lipopolysaccharide virulence determinants. Proc Natl Acad Sci U S A..

[46] Hisatsune K., Kondo S., Isshiki Y., Iguchi T., Haishima Y. (1993). Occurrence of 2-O-methyl-N-(3-deoxy-L-glycero-tetronyl)-D-perosamine (4-amino-4,6-dideoxy-D-manno-pyranose) in lipopolysaccharide from Ogawa but not from Inaba O forms of O1 *Vibrio cholerae*. Biochem Biophys Res Commun.

[47] Gilmour CB. Period of excretion of *Vibrio cholerae* in convalescents. Bull World Health Organ. 1952;7(3):343–51. PMC2554157. PMC2554157 13019553

[48] Mosley WH, Woodward WE, Aziz KM, Rahman AS, Chowdhury AK, Ahmed A, et al. The 1968-1969 cholera-vaccine field trial in rural East Pakistan. Effectiveness of monovalent Ogawa and Inaba vaccines and a purified Inaba antigen, with comparative results of serological and animal protection tests. J Infect Dis. 1970;121:Suppl 121:1-9. PMID: 4912069. 10.1093/infdis/121.supplement.s1 4912069

[49] Gangarosa E.J., Sanati A., Saghari H., Feeley J.C. (1967). Multiple serotypes of *Vibrio cholerae* isolated from a case of cholera Evidence suggesting in-vivo mutation. Lancet.

[50] Bongat A.F.G., Saksena R., Adamo R., Fujimoto Y., Shiokawa Z., Peterson D.C. (2010). Multimeric bivalent immunogens from recombinant tetanus toxin HC fragment, synthetic hexasaccharides, and a glycopeptide adjuvant. Glycoconj J.

[51] Ou L.i., Kong W.-P., Chuang G.-Y., Ghosh M., Gulla K., O’Dell S. (2020). Preclinical development of a fusion peptide conjugate as an HIV vaccine immunogen. Sci Rep.

[52] Maertens K., Burbidge P., Van Damme P., Goldblatt D., Leuridan E. (2017). Pneumococcal immune response in infants whose mothers received tetanus, diphtheria and acellular pertussis vaccination during pregnancy. Pediatr Infect Dis J.

[53] Burns D.L., Kossaczka Z., Lin F.-Y., Ho V.A., Thuy N.T.T., Bay P.V. (1999). Safety and immunogenicity of Vi conjugate vaccines for typhoid fever in adults, teenagers, and 2- to 4-year-old children in Vietnam. Infect Immun.

[54] Cross DL, Verheul MK, Leipold MD, Obermoser G, Jin C, Jones E, et al. Vi-Vaccinations induce heterogeneous plasma cell responses that associate with protection from typhoid fever. Front Immunol. 2020;11:574057. PMC7793947. 10.3389/fimmu.2020.574057 PMC7793947 33424833

[55] Ganguly R, Clem LW, Bencic Z, Sinha R, Sakazaki R, Waldman RH. Antibody response in the intestinal secretions of volunteers immunized with various cholera vaccines. Bull World Health Organ. 1975;52(3):323–30. PMC2366383. PMC2366383 779998

[56] Svennerholm A.M., Holmgren J., Hanson L.A., Lindblad B.S., Quereshi F., Rahimtoola R.J. (1977). Boosting of secretory IgA antibody responses in man by parenteral cholera vaccination. Scand J Immunol.

[57] Mascart-Lemone F., Carlsson B., Jalil F., Hahn-Zoric M., Duchateau J., Hanson L.A. (1988). Polymeric and monomeric IgA response in serum and milk after parenteral cholera and oral typhoid vaccination. Scand J Immunol.

[58] Hahn-Zoric M., Carlsson B., Jalil F., Mellander L., Germanier R., Hanson L.A. (1989). The influence on the secretory IgA antibody levels in lactating women of oral typhoid and parenteral cholera vaccines given alone or in combination. Scand J Infect Dis.

[59] Klugman K.P. (2001). Efficacy of pneumococcal conjugate vaccines and their effect on carriage and antimicrobial resistance. Lancet Infect Dis.

[60] Daugla DM, Gami JP, Gamougam K, Naibei N, Mbainadji L, Narbe M, et al. Effect of a serogroup A meningococcal conjugate vaccine (PsA-TT) on serogroup A meningococcal meningitis and carriage in Chad: a community study [corrected]. Lancet. 2014;383(9911):40-7. PMC3898950. 10.1016/S0140-6736(13)61612-8 PMC3898950 24035220

[61] Takala A.K., Eskola J., Leinonen M., Kayhty H., Nissinen A., Pekkanen E. (1991). Reduction of oropharyngeal carriage of *Haemophilus influenzae* type b (Hib) in children immunized with an Hib conjugate vaccine. J Infect Dis.

[62] Barbour M.L., Booy R., Crook D.W., Griffiths H., Chapel H.M., Moxon E.R. (1993). *Haemophilus influenzae* type b carriage and immunity four years after receiving the *Haemophilus influenzae* oligosaccharide-CRM197 (HbOC) conjugate vaccine. Pediatr Infect Dis J.

[63] Takala A.K., Santosham M., Almeido-Hill J., Wolff M., Newcomer W., Reid R. (1993). Vaccination with *Haemophilus influenzae* type b meningococcal protein conjugate vaccine reduces oropharyngeal carriage of *Haemophilus influenzae* type b among American Indian children. Pediatr Infect Dis J.

[64] Murphy T.V., Pastor P., Medley F., Osterholm M.T., Granoff D.M. (1993). Decreased Haemophilus colonization in children vaccinated with *Haemophilus influenzae* type b conjugate vaccine. J Pediatr.

[65] Safadi MA, Carvalhanas TR, Paula de Lemos A, Gorla MC, Salgado M, Fukasawa LO, et al. Carriage rate and effects of vaccination after outbreaks of serogroup C meningococcal disease, Brazil, 2010. Emerg Infect Dis. 2014;20(5):806-11. PMC4012795. 10.3201/eid2005.130948 PMC4012795 24751156

[66] Clark S.A., Borrow R. (2020). Herd Protection against meningococcal disease through vaccination. Microorganisms..

[67] Clements J.D., Zhang Q., Choo S., Everard J., Jennings R., Finn A. (2000). Mucosal immune responses to meningococcal group C conjugate and group A and C polysaccharide vaccines in adolescents. Infect Immun.

[68] Clements J.D., Zhang Q., Lakshman R., Burkinshaw R., Choo S., Everard J. (2001). Primary and booster mucosal immune responses to meningococcal group A and C conjugate and polysaccharide vaccines administered to university students in the United Kingdom. Infect Immun.

[69] Borrow R., Fox A.J., Cartwright K., Begg N.T., Jones D.M. (1999). Salivary antibodies following parenteral immunization of infants with a meningococcal serogroup A and C conjugated vaccine. Epidemiol Infect.

[70] Nurkka A., MacLennan J., Jantti V., Obaro S., Greenwood B., Kayhty H. (2000). Salivary antibody response to vaccination with meningococcal A/C polysaccharide vaccine in previously vaccinated and unvaccinated Gambian children. Vaccine..

[71] Bårnes G.K., Workalemahu B., Kristiansen P.A., Beyene D., Merdekios B., Fissiha P. (2016). Salivary and serum antibody response against *Neisseria meningitidis* after vaccination with conjugate polysaccharide vaccines in Ethiopian volunteers. Scand J Immunol.

[72] van Ravenhorst MB, den Hartog G, van der Klis FRM, van Rooijen DM, Sanders EAM, Berbers GAM. Induction of salivary antibody levels in Dutch adolescents after immunization with monovalent meningococcal serogroup C or quadrivalent meningococcal serogroup A, C, W and Y conjugate vaccine. PLoS One. 2018;13(4):e0191261. PMC5908077. 10.1371/journal.pone.0191261 PMC5908077 29672552

[73] Sirisinha S., Charupatana C. (1970). Antibody responses in serum, secretions, and urine of man after parenteral administration of vaccines. Infect Immun.

[74] Vianzon V., Illek B., Moe G.R., Burns D.L. (2017). Effect of vaccine-elicited antibodies on colonization of *Neisseria meningitidis* Serogroup B and C strains in a human bronchial epithelial cell culture model. Clin Vaccine Immunol.

[75] Xu P., Alam M.M., Kalsy A., Charles R.C., Calderwood S.B., Qadri F. (2011). Simple, direct conjugation of bacterial O-SP-core antigens to proteins: development of cholera conjugate vaccines. Bioconjug Chem.

