# Supplementary material for: Scalable production and immunogenicity of a cholera conjugate vaccine
Source: Vaccine. 2021 Nov 16;39(47):6936–46. doi: 10.1016/j.vaccine.2021.10.005 (PMC8609181; doi:10.1016/j.vaccine.2021.10.005)
Supplement: Figure S1 — 1H-NMR comparison of OSP produced by Eubiologics (black) with standard (red) [75]. 1H-NMR experiments were measured in D 2 O at 25 ° C in Shigemi NMR tube, at 600 MHz on a Bruker Avance Spectrometer with a TCI cryoprobe. Samples were prepared at ~10 mg/mL concentration. [file mmc1.pptx]

## Slide 1
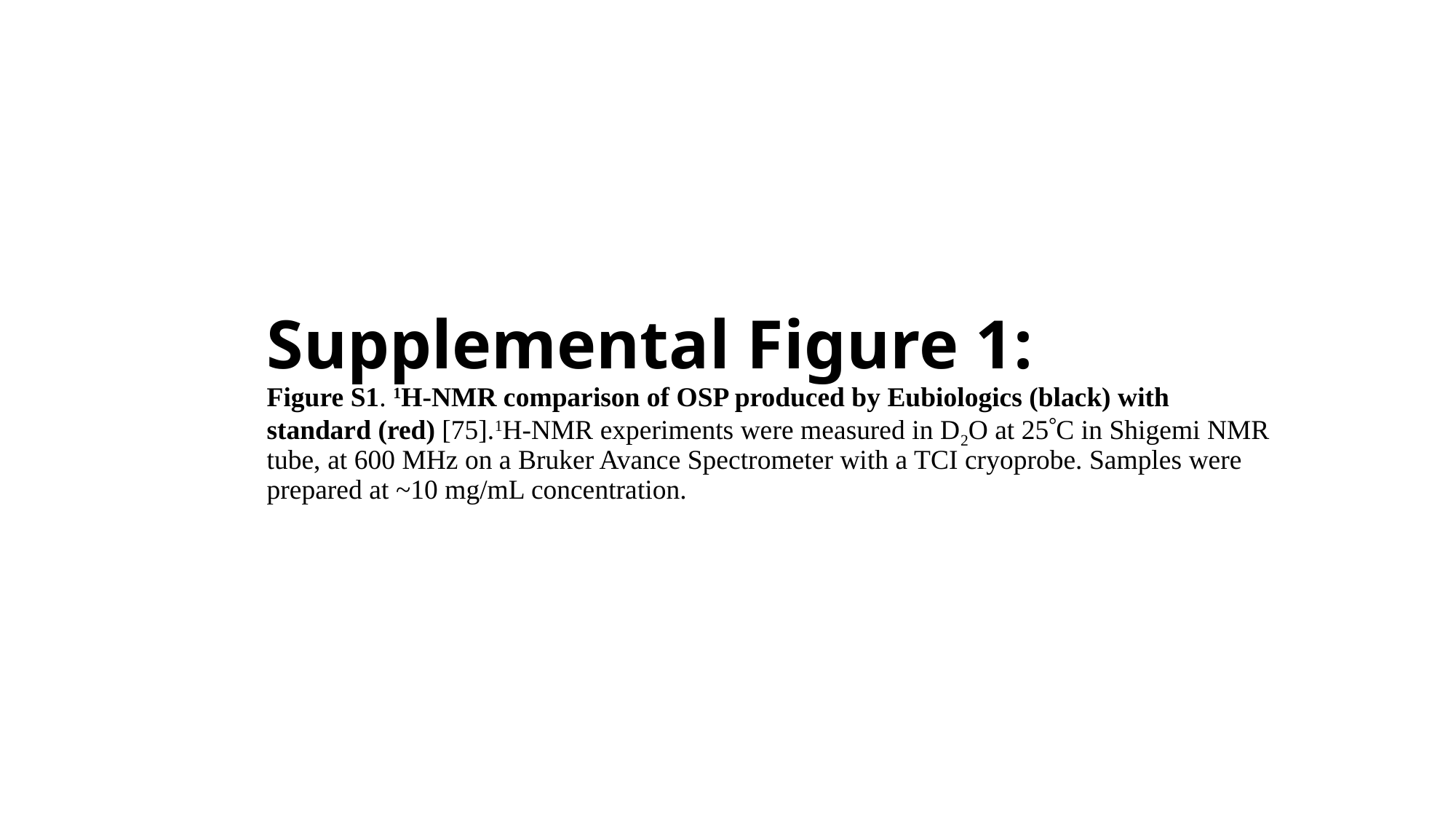

# Supplemental Figure 1: Figure S1. 1H-NMR comparison of OSP produced by Eubiologics (black) with standard (red) [75].1H-NMR experiments were measured in D2O at 25°C in Shigemi NMR tube, at 600 MHz on a Bruker Avance Spectrometer with a TCI cryoprobe. Samples were prepared at ~10 mg/mL concentration.

## Slide 2
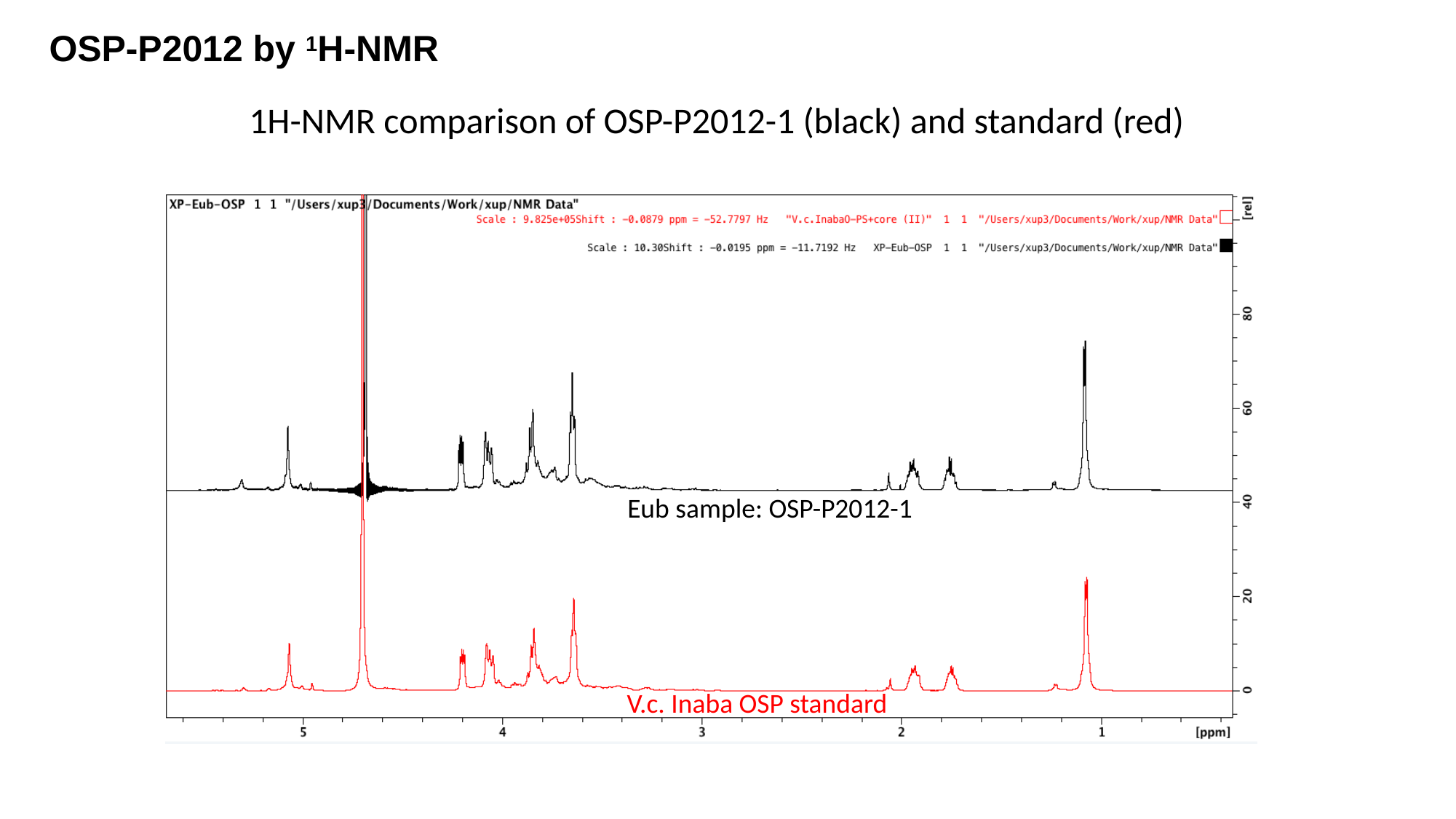

OSP-P2012 by 1H-NMR
1H-NMR comparison of OSP-P2012-1 (black) and standard (red)
Eub sample: OSP-P2012-1
V.c. Inaba OSP standard
