# Supplementary material for: Scalable production and immunogenicity of a cholera conjugate vaccine
Source: Vaccine. 2021 Nov 16;39(47):6936–46. doi: 10.1016/j.vaccine.2021.10.005 (PMC8609181; doi:10.1016/j.vaccine.2021.10.005)
Supplement: Figure S3 — Purified rTTHc analyzed by SE-HPLC compared with standard . rTTHc was analyzed using TSKgel G2000SWxL column on Waters HPLC system at 25° C, eluted with 10 mM sodium phosphate, 150 mM NaCl, pH 7.2) at 0.5 mL/min flow rate. [file mmc3.pptx]

## Slide 1
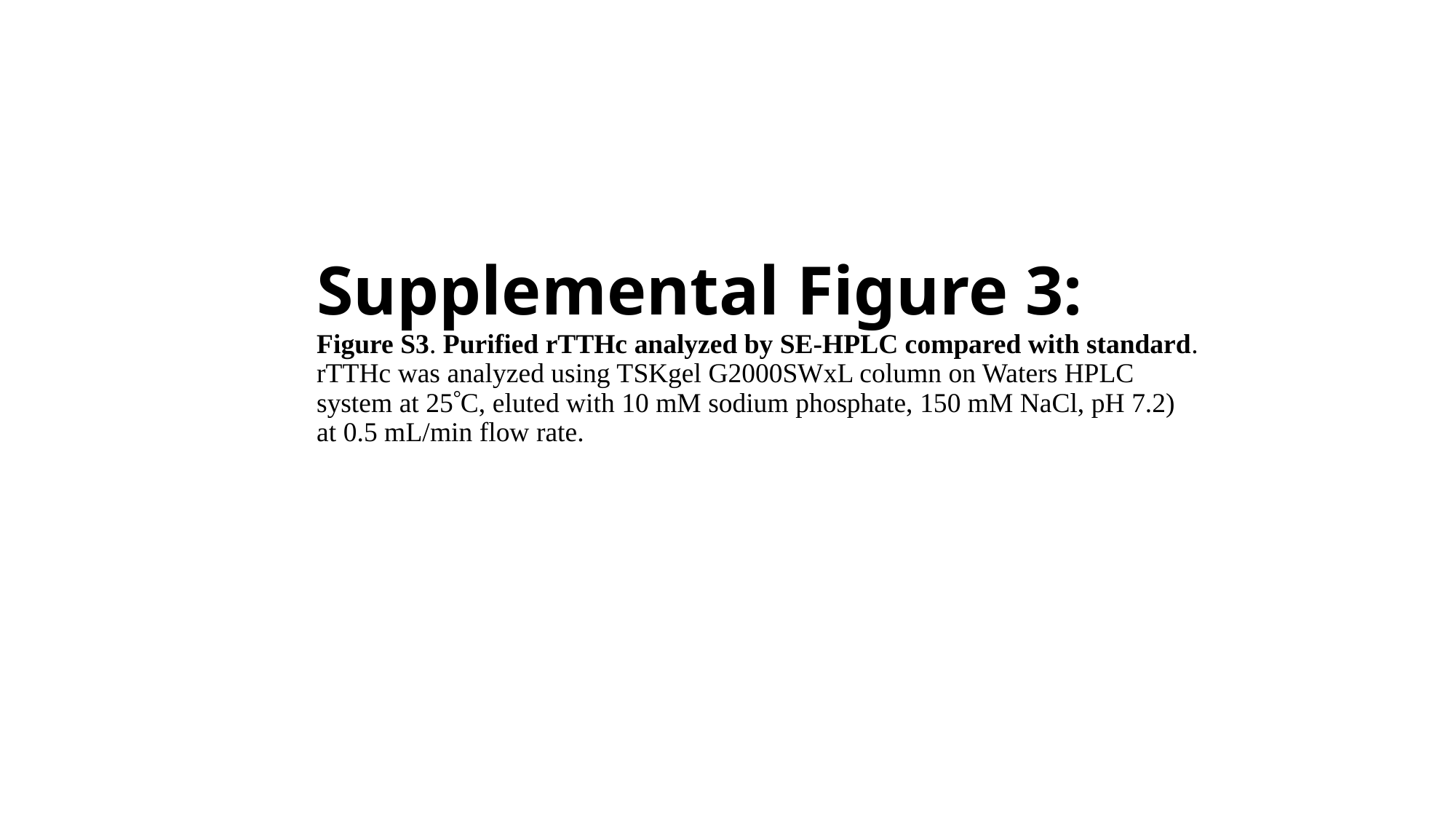

# Supplemental Figure 3: Figure S3. Purified rTTHc analyzed by SE-HPLC compared with standard. rTTHc was analyzed using TSKgel G2000SWxL column on Waters HPLC system at 25°C, eluted with 10 mM sodium phosphate, 150 mM NaCl, pH 7.2) at 0.5 mL/min flow rate.

## Slide 2
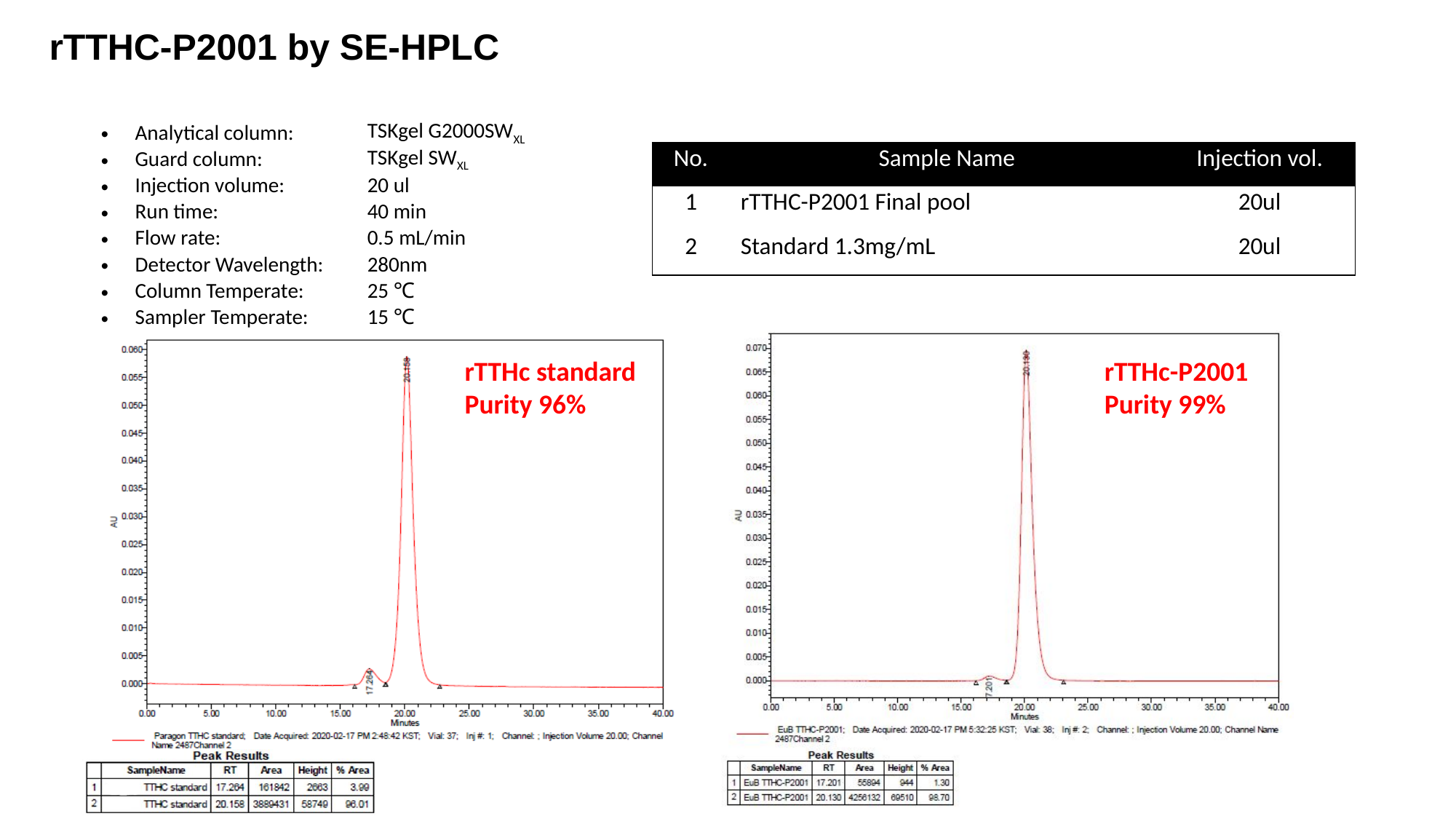

rTTHC-P2001 by SE-HPLC
| Analytical column: | TSKgel G2000SWXL |
| --- | --- |
| Guard column: | TSKgel SWXL |
| Injection volume: | 20 ul |
| Run time: | 40 min |
| Flow rate: | 0.5 mL/min |
| Detector Wavelength: | 280nm |
| Column Temperate: | 25 ℃ |
| Sampler Temperate: | 15 ℃ |
| No. | Sample Name | Injection vol. |
| --- | --- | --- |
| 1 | rTTHC-P2001 Final pool | 20ul |
| 2 | Standard 1.3mg/mL | 20ul |
rTTHc standard
Purity 96%
rTTHc-P2001
Purity 99%
