# Supplementary material for: Scalable production and immunogenicity of a cholera conjugate vaccine
Source: Vaccine. 2021 Nov 16;39(47):6936–46. doi: 10.1016/j.vaccine.2021.10.005 (PMC8609181; doi:10.1016/j.vaccine.2021.10.005)

## Slide 1
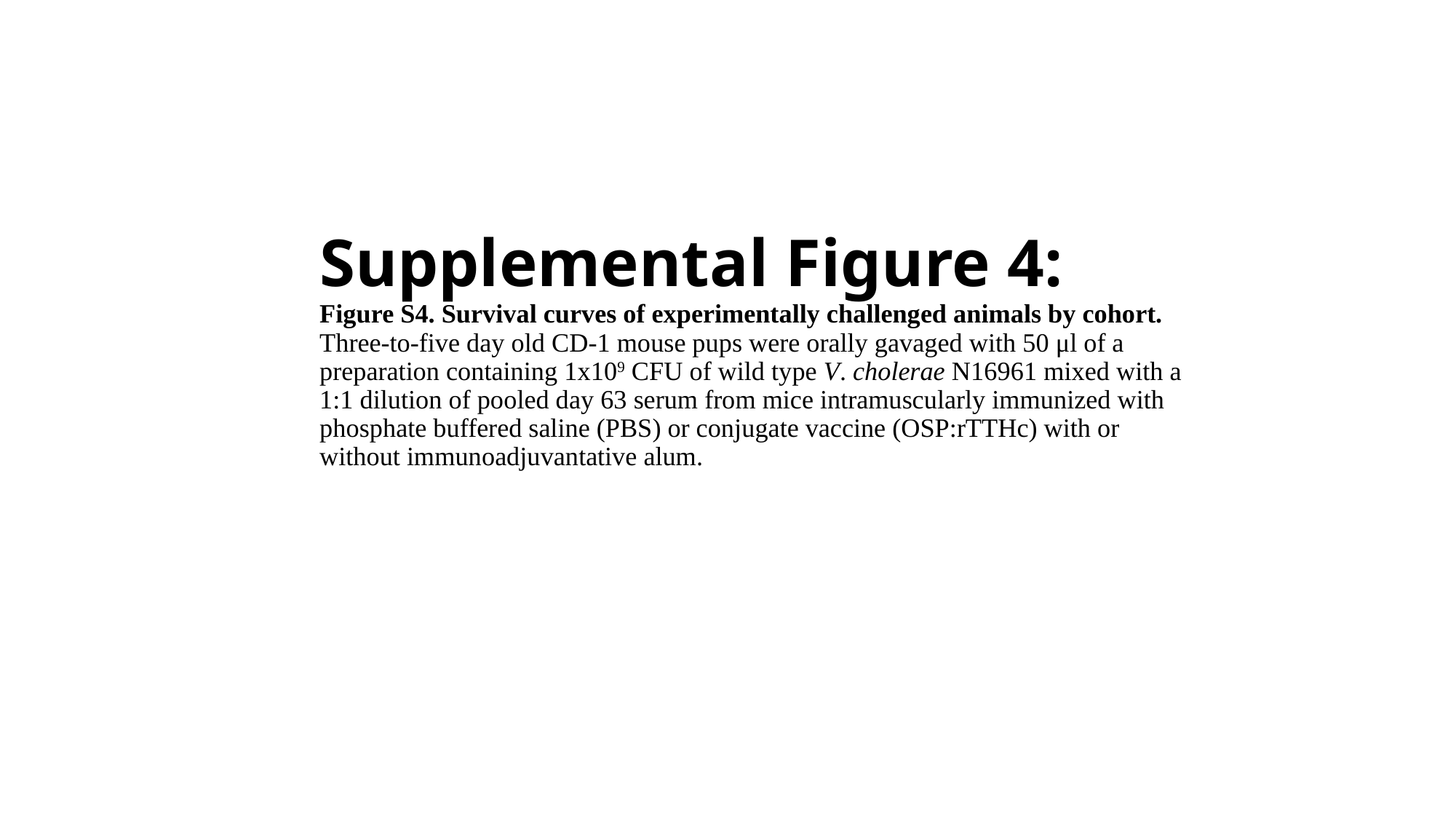

# Supplemental Figure 4: Figure S4. Survival curves of experimentally challenged animals by cohort. Three-to-five day old CD-1 mouse pups were orally gavaged with 50 μl of a preparation containing 1x10^9^ CFU of wild type V. cholerae N16961 mixed with a 1:1 dilution of pooled day 63 serum from mice intramuscularly immunized with phosphate buffered saline (PBS) or conjugate vaccine (OSP:rTTHc) with or without immunoadjuvantative alum.

## Slide 2
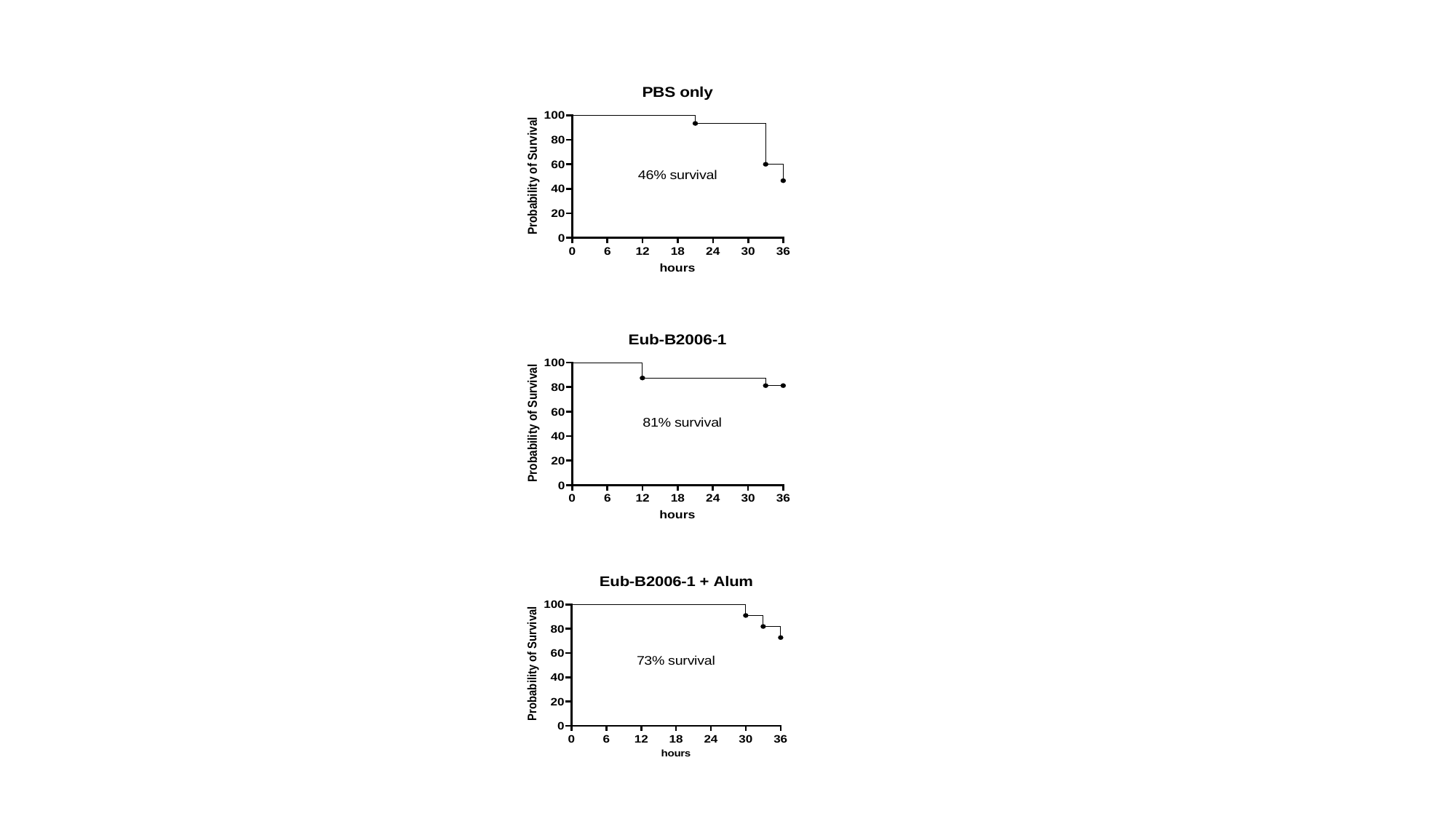

Supplement: Figure S4 — Survival curves of experimentally challenged animals by cohort. Three-to-five day old CD-1 mouse pups were orally gavaged with 50 μl of a preparation containing 1x10^9^ CFU of wild type V. cholerae N16961 mixed with a 1:1 dilution of pooled day 63 serum from mice intramuscularly immunized with phosphate buffered saline (PBS) or conjugate vaccine ( OSP:rTTHc ) with or without immunoadjuvantative alum. [file mmc4.pptx]
