# Supplementary material for: Scalable production and immunogenicity of a cholera conjugate vaccine
Source: Vaccine. 2021 Nov 16;39(47):6936–46. doi: 10.1016/j.vaccine.2021.10.005 (PMC8609181; doi:10.1016/j.vaccine.2021.10.005)

## Slide 1
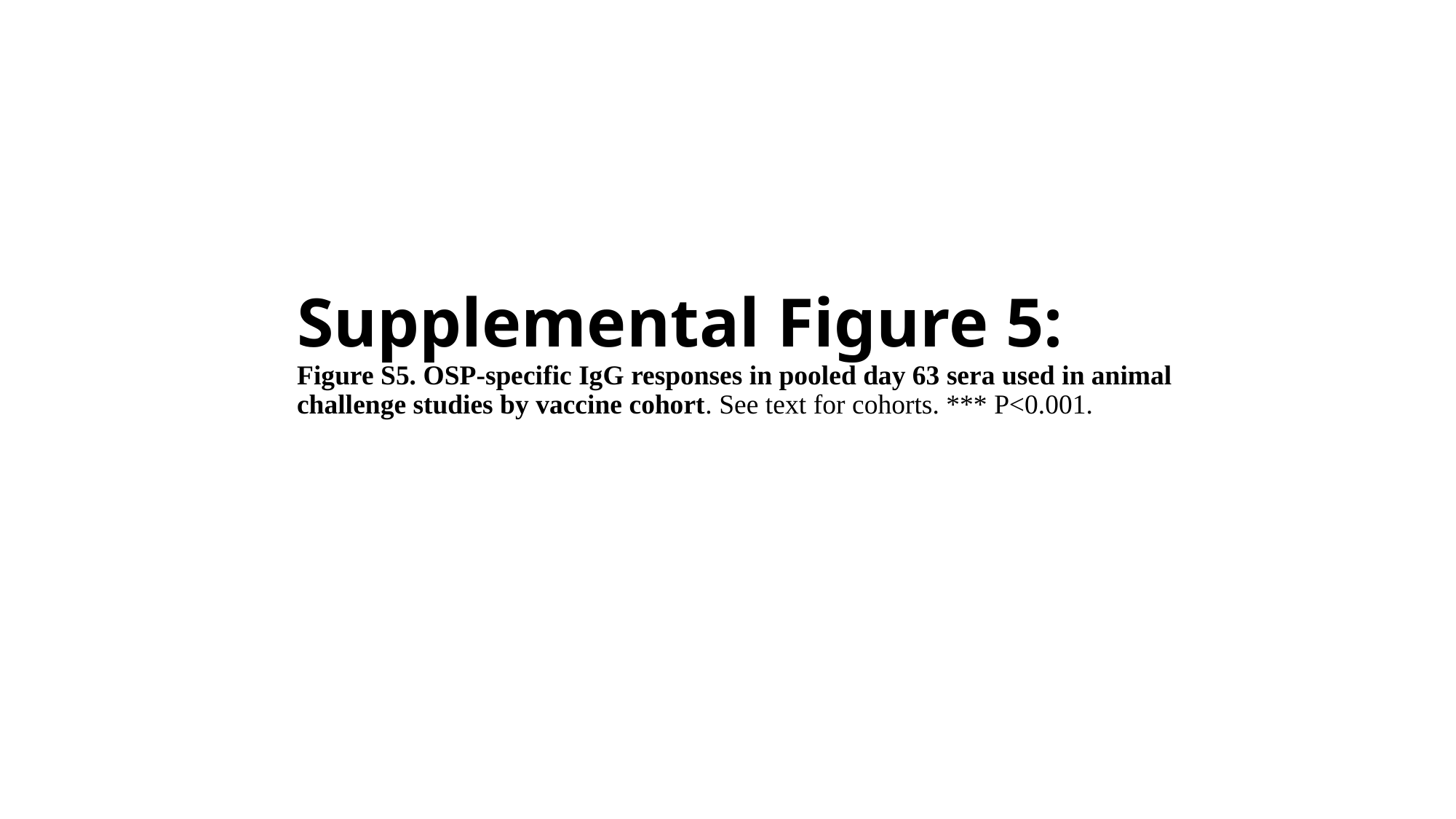

# Supplemental Figure 5: Figure S5. OSP-specific IgG responses in pooled day 63 sera used in animal challenge studies by vaccine cohort. See text for cohorts. *** P<0.001.

## Slide 2
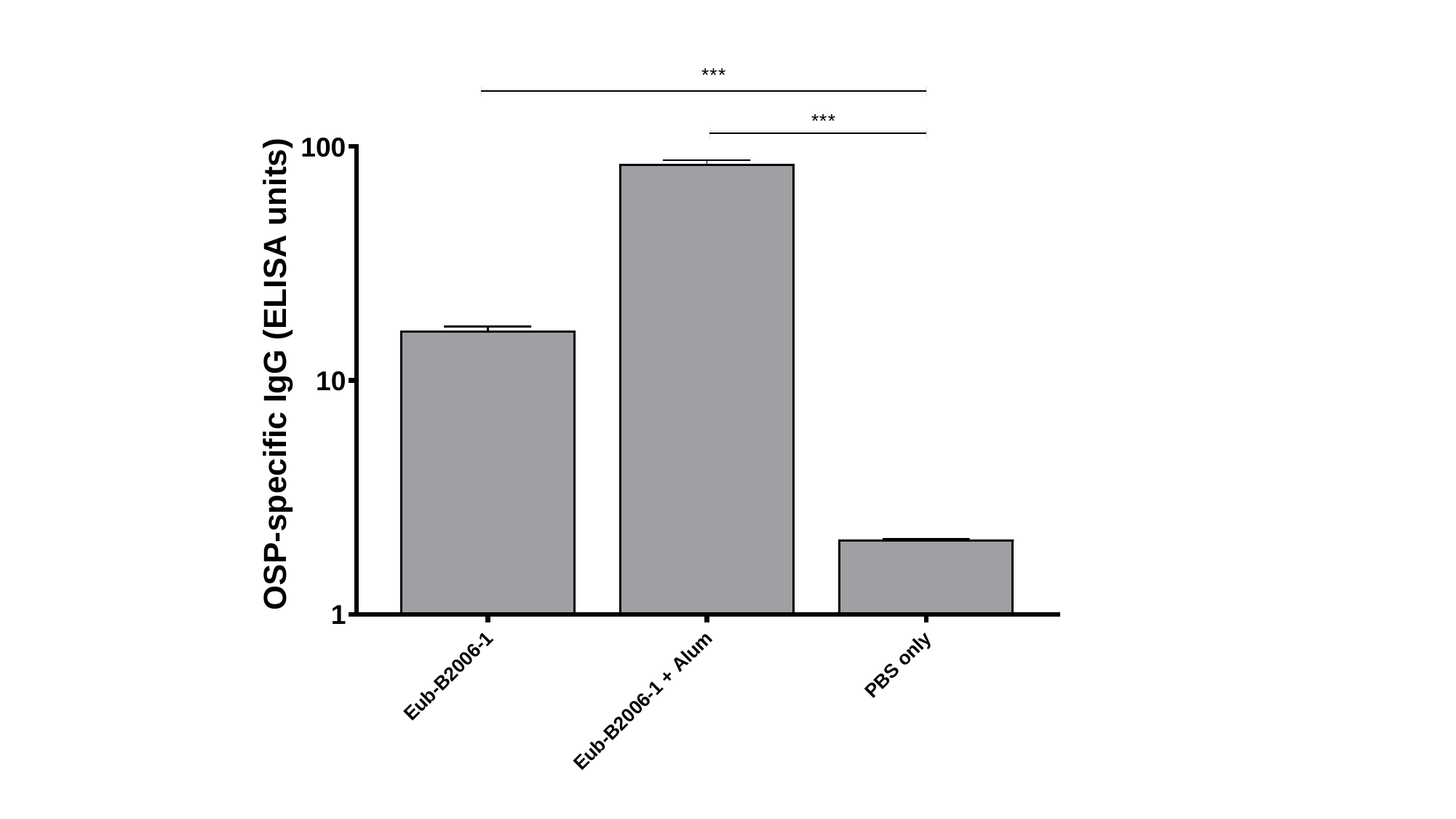

Supplement: Figure S5 — OSP-specific IgG responses in pooled day 63 sera used in animal challenge studies by vaccine cohort . See text for cohorts. *** P<0.001. [file mmc5.pptx]
