# Supplementary material for: Scalable production and immunogenicity of a cholera conjugate vaccine
Source: Vaccine. 2021 Nov 16;39(47):6936–46. doi: 10.1016/j.vaccine.2021.10.005 (PMC8609181; doi:10.1016/j.vaccine.2021.10.005)

**Supplemental Tables**

**Responder Frequencies**

Comparisons by day (d) and by vaccine cohort; Chi2. Column values represent number of responders in each cohort at each time point.

See Figures 5-9.

| **Responder Frequency OSP IgG** | | | |
| --- | --- | --- | --- |
|  | **Alum** | **B2006-1 + Alum** | **Chi2** |
| d0 | 0 | 0 | N/A |
| d7 | 0 | 0 | N/A |
| d14 | 0 | 2 | 0.389 |
| d21 | 0 | 8 | **0.035** |
| d28 | 0 | 9 | **0.019** |
| d35 | 0 | 13 | **0.0004** |
| d56 | 0 | 13 | **0.0004** |
| d63 | 0 | 14 | **<0.0001** |
|  |  |  |  |
|  |  |  |  |
|  | **PBS** | **B2006-1** | **Chi2** |
| d0 | 0 | 0 | N/A |
| d7 | 0 | 0 | N/A |
| d14 | 0 | 1 | 0.5536 |
| d21 | 0 | 2 | 0.389 |
| d28 | 0 | 2 | 0.389 |
| d35 | 0 | 5 | 0.136 |
| d56 | 0 | 6 | 0.091 |
| d63 | 0 | 9 | **0.019** |
|  |  |  |  |
|  |  |  |  |
|  | **B2006-1** | **B2006-2** | **Chi2** |
| d0 | 0 | 0 | N/A |
| d7 | 0 | 0 | N/A |
| d14 | 1 | 0 | 0.309 |
| d21 | 2 | 0 | 0.143 |
| d28 | 2 | 2 | N/A |
| d35 | 5 | 6 | 0.704 |
| d56 | 6 | 6 | N/A |
| d63 | 9 | 6 | 0.273 |
|  |  |  |  |
|  |  |  |  |
|  | **B2006-1** | **B2006-1 + Alum** | **Chi2** |
| d0 | 0 | 0 | N/A |
| d7 | 0 | 0 | N/A |
| d14 | 1 | 2 | 0.542 |
| d21 | 2 | 8 | **0.02** |
| d28 | 2 | 9 | **0.008** |
| d35 | 5 | 13 | **0.0029** |
| d56 | 6 | 13 | **0.008** |
| d63 | 9 | 14 | **0.03** |

| **Responder Frequency OSP IgM** | | | |
| --- | --- | --- | --- |
|  | **Alum** | **B2006-1 + Alum** | **Chi2** |
| d0 | 0 | 0 | N/A |
| d7 | 0 | 2 | 0.389 |
| d14 | 0 | 3 | 0.278 |
| d21 | 0 | 8 | **0.035** |
| d28 | 0 | 3 | 0.278 |
| d35 | 1 | 9 | 0.121 |
| d56 | 1 | 4 | 0.765 |
| d63 | 0 | 7 | 0.058 |
|  |  |  |  |
|  |  |  |  |
|  | **PBS** | **B2006-1** | **Chi2** |
| d0 | 0 | 0 | N/A |
| d7 | 0 | 0 | N/A |
| d14 | 0 | 0 | N/A |
| d21 | 0 | 2 | 0.389 |
| d28 | 0 | 3 | 0.278 |
| d35 | 0 | 8 | **0.035** |
| d56 | 0 | 5 | 0.136 |
| d63 | 0 | 6 | 0.091 |
|  |  |  |  |
|  |  |  |  |
|  | **B2006-1** | **B2006-2** | **Chi2** |
| d0 | 0 | 0 | N/A |
| d7 | 0 | 0 | N/A |
| d14 | 0 | 1 | 0.309 |
| d21 | 2 | 5 | 0.195 |
| d28 | 3 | 5 | 0.409 |
| d35 | 8 | 6 | 0.464 |
| d56 | 5 | 4 | 0.69 |
| d63 | 6 | 8 | 0.464 |
|  |  |  |  |
|  |  |  |  |
|  | **B2006-1** | **B2006-1 + Alum** | **Chi2** |
| d0 | 0 | 0 | N/A |
| d7 | 0 | 2 | 0.143 |
| d14 | 0 | 3 | 0.067 |
| d21 | 2 | 8 | 0.2 |
| d28 | 3 | 3 | N/A |
| d35 | 8 | 9 | 0.712 |
| d56 | 5 | 4 | 0.69 |
| d63 | 6 | 7 | 0.712 |

| **Responder Frequency LPS IgG** | | | |
| --- | --- | --- | --- |
|  | **Alum** | **B2006-1 + Alum** | **Chi2** |
| d0 | 0 | 0 | N/A |
| d7 | 0 | 0 | N/A |
| d14 | 0 | 0 | N/A |
| d21 | 0 | 2 | 0.389 |
| d28 | 0 | 3 | 0.278 |
| d35 | 0 | 6 | 0.091 |
| d56 | 0 | 6 | 0.091 |
| d63 | 0 | 8 | **0.035** |
|  |  |  |  |
|  |  |  |  |
|  | **PBS** | **B2006-1** | **Chi2** |
| d0 | 0 | 0 | N/A |
| d7 | 0 | 0 | N/A |
| d14 | 0 | 0 | N/A |
| d21 | 0 | 0 | N/A |
| d28 | 0 | 0 | N/A |
| d35 | 0 | 0 | N/A |
| d56 | 0 | 1 | 0.5536 |
| d63 | 0 | 5 | 0.136 |
|  |  |  |  |
|  |  |  |  |
|  | **B2006-1** | **B2006-2** | **Chi2** |
| d0 | 0 | 0 | N/A |
| d7 | 0 | 0 | N/A |
| d14 | 0 | 0 | N/A |
| d21 | 0 | 0 | N/A |
| d28 | 0 | 0 | N/A |
| d35 | 0 | 0 | N/A |
| d56 | 1 | 1 | N/A |
| d63 | 5 | 4 | 0.69 |
|  |  |  |  |
|  |  |  |  |
|  | **B2006-1** | **B2006-1 + Alum** | **Chi2** |
| d0 | 0 | 0 | N/A |
| d7 | 0 | 0 | N/A |
| d14 | 0 | 0 | N/A |
| d21 | 0 | 2 | 0.143 |
| d28 | 0 | 3 | 0.067 |
| d35 | 0 | 6 | **0.006** |
| d56 | 1 | 6 | **0.03** |
| d63 | 5 | 8 | 0.269 |

| **Responder Frequency LPS IgM** | | | |
| --- | --- | --- | --- |
|  | **Alum** | **B2006-1 + Alum** | **Chi2** |
| d0 | 0 | 0 | N/A |
| d7 | 0 | 1 | 0.5536 |
| d14 | 0 | 2 | 0.389 |
| d21 | 0 | 1 | 0.5536 |
| d28 | 0 | 0 | N/A |
| d35 | 0 | 3 | 0.278 |
| d56 | 0 | 2 | 0.389 |
| d63 | 0 | 6 | 0.091 |
|  |  |  |  |
|  |  |  |  |
|  | **PBS** | **B2006-1** | **Chi2** |
| d0 | 0 | 0 | N/A |
| d7 | 0 | 1 | 0.5536 |
| d14 | 0 | 0 | N/A |
| d21 | 0 | 1 | 0.5536 |
| d28 | 0 | 0 | N/A |
| d35 | 0 | 2 | 0.389 |
| d56 | 0 | 1 | 0.5536 |
| d63 | 0 | 4 | 0.196 |
|  |  |  |  |
|  |  |  |  |
|  | **B2006-1** | **B2006-2** | **Chi2** |
| d0 | 0 | 0 | N/A |
| d7 | 1 | 2 | 0.542 |
| d14 | 0 | 0 | N/A |
| d21 | 1 | 1 | N/A |
| d28 | 0 | 2 | 0.143 |
| d35 | 2 | 3 | 0.624 |
| d56 | 1 | 2 | 0.542 |
| d63 | 4 | 7 | 0.255 |
|  |  |  |  |
|  |  |  |  |
|  | **B2006-1** | **B2006-1 + Alum** | **Chi2** |
| d0 | 0 | 0 | N/A |
| d7 | 1 | 1 | N/A |
| d14 | 0 | 2 | 0.143 |
| d21 | 1 | 1 | N/A |
| d28 | 0 | 0 | N/A |
| d35 | 2 | 3 | 0.624 |
| d56 | 1 | 2 | 0.542 |
| d63 | 4 | 6 | 0.438 |

| **Responder Frequency rTTHc IgG** | | | |
| --- | --- | --- | --- |
|  | **Alum** | **B2006-1 + Alum** | **Chi2** |
| d0 | 0 | 0 | N/A |
| d7 | 3 | 4 | 0.176 |
| d14 | 0 | 14 | **<0.0001** |
| d21 | 0 | 15 | **<0.0001** |
| d28 | 0 | 15 | **<0.0001** |
| d35 | 1 | 15 | **0.0001** |
| d56 | 0 | 15 | **<0.0001** |
| d63 | 0 | 15 | **<0.0001** |
|  |  |  |  |
|  |  |  |  |
|  | **PBS** | **B2006-1** | **Chi2** |
| d0 | 0 | 0 | N/A |
| d7 | 2 | 0 | **0.0098** |
| d14 | 0 | 8 | **0.035** |
| d21 | 1 | 15 | **0.0001** |
| d28 | 0 | 14 | **<0.0001** |
| d35 | 2 | 14 | **0.0098** |
| d56 | 0 | 14 | **<0.0001** |
| d63 | 0 | 15 | **<0.0001** |
|  |  |  |  |
|  |  |  |  |
|  | **B2006-1** | **B2006-2** | **Chi2** |
| d0 | 0 | 0 | N/A |
| d7 | 0 | 0 | N/A |
| d14 | 8 | 7 | 0.71 |
| d21 | 15 | 13 | 0.14 |
| d28 | 14 | 12 | 0.282 |
| d35 | 14 | 15 | 0.309 |
| d56 | 14 | 14 | N/A |
| d63 | 15 | 13 | 0.14 |
|  |  |  |  |
|  |  |  |  |
|  | **B2006-1** | **B2006-1 + Alum** | **Chi2** |
| d0 | 0 | 0 | N/A |
| d7 | 0 | 4 | **0.031** |
| d14 | 8 | 14 | **0.013** |
| d21 | 15 | 15 | N/A |
| d28 | 14 | 15 | 0.309 |
| d35 | 14 | 15 | 0.309 |
| d56 | 14 | 15 | 0.309 |
| d63 | 15 | 15 | N/A |

| **Responder Frequency rTTHc IgM** | | | |
| --- | --- | --- | --- |
|  | **Alum** | **B2006-1 + Alum** | **Chi2** |
| d0 | 0 | 0 | N/A |
| d7 | 0 | 4 | 0.196 |
| d14 | 0 | 0 | N/A |
| d21 | 0 | 10 | **0.0098** |
| d28 | 0 | 4 | 0.196 |
| d35 | 0 | 1 | 0.5536 |
| d56 | 0 | 0 | N/A |
| d63 | 0 | 2 | 0.389 |
|  |  |  |  |
|  |  |  |  |
|  | **PBS** | **B2006-1** | **Chi2** |
| d0 | 0 | 3 | 0.278 |
| d7 | 0 | 2 | 0.389 |
| d14 | 0 | 0 | N/A |
| d21 | 1 | 4 | 0.765 |
| d28 | 2 | 4 | 0.573 |
| d35 | 1 | 5 | 0.573 |
| d56 | 0 | 0 | N/A |
| d63 | 0 | 0 | N/A |
|  |  |  |  |
|  |  |  |  |
|  | **B2006-1** | **B2006-2** | **Chi2** |
| d0 | 3 | 3 | N/A |
| d7 | 2 | 3 | 0.624 |
| d14 | 0 | 1 | 0.309 |
| d21 | 4 | 6 | 0.438 |
| d28 | 4 | 5 | 0.69 |
| d35 | 5 | 7 | 0.456 |
| d56 | 0 | 0 | N/A |
| d63 | 0 | 2 | 0.143 |
|  |  |  |  |
|  |  |  |  |
|  | **B2006-1** | **B2006-1 + Alum** | **Chi2** |
| d0 | 3 | 0 | 0.067 |
| d7 | 2 | 4 | 0.361 |
| d14 | 0 | 0 | N/A |
| d21 | 4 | 10 | **0.028** |
| d28 | 4 | 4 | N/A |
| d35 | 5 | 1 | 0.067 |
| d56 | 0 | 0 | N/A |
| d63 | 0 | 2 | 0.143 |


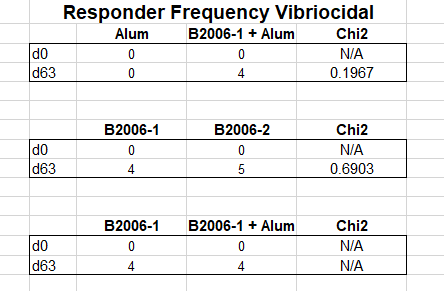


**Responder Frequency OSP IgG Memory B cells in spleen day 63**


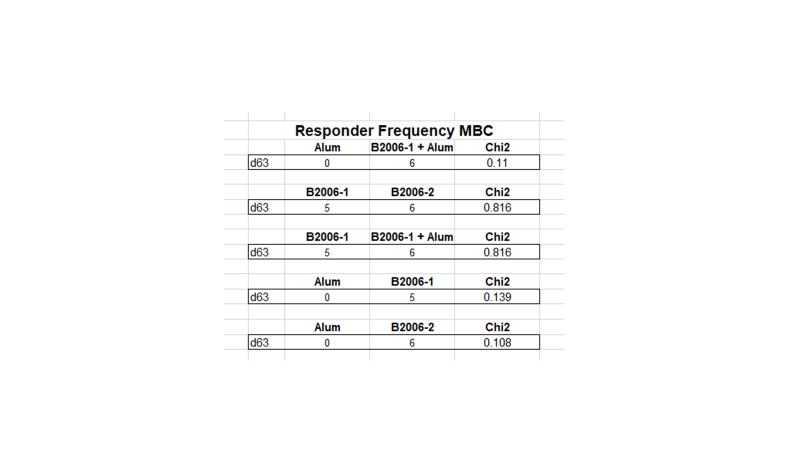

Supplement: Supplementary Tables [file mmc6.docx]
